# The impact of therapeutic radiation on drug distribution across the blood-brain barrier in normal mouse brain and orthotopic glioblastoma tumors

**DOI:** 10.1093/neuonc/noaf093

**Published:** 2025-10-14

**Authors:** Wenjuan Zhang, Michael P. Grams, Rajneet K. Oberoi, Ju-Hee Oh, Paul A. Decker, Terence T. Sio, Surabhi Talele, Zachary C. Wilson, Margaret A. Connors, Katrina K. Bakken, Brett L. Carlson, Lauren L. Ott, Danielle M. Burgenske, Erik J. Tryggestad, Jeanette E. Eckel Passow, William F. Elmquist, Jann N. Sarkaria

**Affiliations:** Brain Barriers Research Center, Department of Pharmaceutics, College of Pharmacy, University of Minnesota, Minneapolis, Minnesota, USA; Department of Radiation Oncology, Mayo Clinic, Rochester, Minnesota, USA; Brain Barriers Research Center, Department of Pharmaceutics, College of Pharmacy, University of Minnesota, Minneapolis, Minnesota, USA; Brain Barriers Research Center, Department of Pharmaceutics, College of Pharmacy, University of Minnesota, Minneapolis, Minnesota, USA; Department of Radiation Oncology, Mayo Clinic, Rochester, Minnesota, USA; Department of Radiation Oncology, Mayo Clinic, Rochester, Minnesota, USA; Brain Barriers Research Center, Department of Pharmaceutics, College of Pharmacy, University of Minnesota, Minneapolis, Minnesota, USA; Department of Radiation Oncology, Mayo Clinic, Rochester, Minnesota, USA; Department of Radiation Oncology, Mayo Clinic, Rochester, Minnesota, USA; Department of Radiation Oncology, Mayo Clinic, Rochester, Minnesota, USA; Department of Radiation Oncology, Mayo Clinic, Rochester, Minnesota, USA; Department of Radiation Oncology, Mayo Clinic, Rochester, Minnesota, USA; Department of Radiation Oncology, Mayo Clinic, Rochester, Minnesota, USA; Department of Radiation Oncology, Mayo Clinic, Rochester, Minnesota, USA; Department of Radiation Oncology, Mayo Clinic, Rochester, Minnesota, USA; Brain Barriers Research Center, Department of Pharmaceutics, College of Pharmacy, University of Minnesota, Minneapolis, Minnesota, USA; Department of Radiation Oncology, Mayo Clinic, Rochester, Minnesota, USA

**Keywords:** BBB permeability, brain tumor models, drug delivery, radiation therapy

## Abstract

**Background.:**

Most oncology therapeutics have limited distribution into the brain, and developing strategies to overcome this limitation would be clinically impactful. While therapeutic radiation is often cited as a strategy to accomplish this, there are no published studies demonstrating the effect of radiation on drug distribution into the brain or brain tumors.

**Methods.:**

Mice were treated with brain radiation (6 Gy × 5, 4 Gy × 10; 40 Gy × 1) and dosed with drugs (levetiracetam, cefazolin, nedisertib, brigimadlin, apitolisib, or GNE-317) at times ranging from just prior to months after radiation. Plasma and tissue drug concentrations were measured by LC-MS/MS.

**Results.:**

Radiation did not significantly enhance drug delivery into brain tissue for levetiracetam, cefazolin, GNE-317, apitolisib, or nedisertib at any time post-radiation. Even a single, supra-therapeutic dose of radiation (40 Gy) did not significantly affect brain distribution of GNE-317 or apitolisib (*P* ≥ .07) from 16 to 160 hours post-radiation. For brigimadlin, radiation (6 Gy × 5) was associated with a modest but significant increase in drug accumulation only at 72 hours post-radiation (brain-to-plasma ratio 0.014 ± 0.006 vs. 0.025 ± 0.010, respectively; *P* = .04), but not at any other timepoint (24 hours, 15, 28, 94, 133, 183 days; *P* > .05). Similarly, radiation (6 Gy × 5) of orthotopic tumors did not increase levels of brigimadlin in GBM10 or GBM108 or nedisertib in GBM108 (*P* > .05).

**Conclusions.:**

Radiation had no meaningful impact on drug delivery into brain or brain tumors for the drugs tested.

The blood-brain barrier (BBB) is important for protecting the central nervous system (CNS) from pathogens and toxins. Connections between brain endothelial cells, pericytes, and astrocytic endfeet promote continuous tight junctions between endothelial cells and physically prevent paracellular diffusion of molecules into the brain parenchyma. As a result, most substances enter the brain through diffusion or facilitated transport across capillary endothelial cell membranes, and the activity of efflux transporters on the luminal capillary membranes significantly limits CNS distribution for efflux substrates.^1^ The BBB also is intact in almost all gliomas and early secondary brain metastatic lesions. These physical and biochemical barriers limit the CNS distribution and efficacy for the vast majority of drugs used in oncology.^2–6^ Thus, developing strategies to disrupt the BBB can be an important strategy for developing curative strategies for both primary and secondary brain tumors.^7^

Radiation therapy is a cornerstone of therapy for brain tumors and can alter the integrity of the BBB. A typical radiation regimen for glioblastoma (GBM), the most common high-grade glioma, is partial brain irradiation to a dose of 60 Gy in 30 radiation treatments (fractions) or 40 Gy in 15 fractions for elderly patients.^8^ Brain metastatic disease is treated either with radiosurgery, with radiation focused only on visible tumors to a dose of 14 to 30 Gy delivered in 1 to 5 fractions, or with whole brain radiation to a dose of 30 Gy in 10 fractions. Extensive preclinical work in animal models has demonstrated the acute effects of radiation on the BBB with evidence for induction of apoptosis and senescence of endothelial cells,^2,9^ reduction in tight junction proteins,^10^ and altered efflux transporter activity within the microvasculature.^2,10–12^ In patients, disruption of the BBB following radiation has been demonstrated as radiationinduced changes in the accumulation of radiographic contrast material, which is normally not brain penetrant.^13,14^ While these studies demonstrate that radiation affects the physiology of the BBB, there has not been a careful evaluation of how radiation impacts the distribution of therapeutic agents into the CNS.

The present study evaluates the acute and chronic impact of therapeutically relevant radiation exposures on drug distribution in the brains of normal mice and GBM patient-derived xenografts (PDXs). While subtle effects were observed within days of radiation, the effects on drug distribution were small, transient, and unlikely to be clinically meaningful.

## Materials and Methods

### Drug Formulations and Dosing

Six drugs with varying BBB permeabilities were used. Levetiracetam and cefazolin (Hikma Farmaceutica) were co-administered intraperitoneally to mice. Brigimadlin (BI-907828, supplied by Boehringer Ingelheim) was formulated in 0.5% Natrosol^™^ for oral gavage. Nedisertib (M3814; supplied by the National Cancer Institute) was suspended in 0.25% hydroxypropyl methylcellulose and 0.25% Tween 20 in sodium citrate buffer for oral administration. GNE-317 and apitolisib (supplied by Genentech) were suspended in 0.5% methylcellulose with 0.2% Tween 80 for oral co-administration.

### Study Design

The impact of radiation on drug distribution into the brain was investigated through experiments examining acute, subacute, and chronic effects on CNS distribution of drugs with various BBB permeabilities (Figure 1). Additional details for these studies are provided in Supplementary Methods.

#### Study 1: acute effects of radiation on BBB permeability of levetiracetam and cefazolin.—

Evaluated in FVB mice treated with sham or hemi-brain radiation (30 Gy in 5 fractions). Levetiracetam (8.4 mg/kg) and cefazolin (50 mg/kg) were co-dosed 15 minutes after the last radiation treatment. Mice were euthanized at 15 minutes or 1 hour post-dose.

#### Study 2: acute and subacute effects of radiation on BBB permeability of GNE-317 and apitolisib.—

Evaluated in C57BL/6 mice treated with sham or hemi-brain irradiation (40 Gy in 10 fractions). GNE-317 (30 mg/kg) and apitolisib (7.5 mg/kg) were co-dosed at various time points post-radiation. Mice were euthanized at 1 hour post-dose.

#### Study 3: acute effects of single high-dose radiation on BBB permeability of GNE-317 and apitolisib.—

Evaluated in C57BL/6 mice treated with a single 40 Gy fraction of hemi-brain radiation. GNE-317 (30 mg/kg) and apitolisib (7.5 mg/kg) were orally co-dosed at various time points post-radiation. Mice were euthanized at 1 hour post-dose.

#### Study 4: acute effects of radiation on BBB permeability of nedisertib.—

Evaluated in FVB mice treated with hemi-brain irradiation (30 Gy in 5 fractions). Nedisertib (60 mg/kg) was orally dosed 10 minutes after the last radiation treatment. Mice were euthanized at 2 or 5 hours post-dose.

#### Study 5: acute, subacute, and chronic effects of radiation on BBB permeability of brigimadlin.—

Evaluated in FVB mice treated with hemi-brain irradiation (30 Gy in 5 fractions). Brigimadlin (10 mg/kg) was orally dosed at various time points post-radiation. Mice were euthanized at 24 hours post-dose.

#### Study 6: acute effects of radiation on BBB permeability in PDX brain tumor models.—

Evaluated in GBM108-eGFPfLuc2 and GBM10 orthotopic xenografts. Mice were treated with sham or supratentorial irradiation (30 Gy in 5 fractions). Brigimadlin (10 mg/kg) or nedisertib (60 mg/kg) were orally dosed immediately before the last radiation dose. Mice were euthanized at 24 or 2 hours post-dose of brigimadlin or nedisertib, respectively.

### Mouse Radiation Setup

Radiation was delivered under general anesthesia using a stereotactic bite block. Irradiation for studies 1 and 4–6 was performed using the X-RAD SmART irradiator with integrated CBCT.^15^ For hemi-brain irradiation, a single posterior-anterior beam was delivered using a 20 mm square collimator, with the lateral edge of the beam setup along the midline of the head to deliver radiation only to the right side of the brain. For supratentorial irradiation, a 10 mm circular beam was delivered via opposed lateral beams. Before the SmART irradiator was available, mice in studies 2 and 3 were irradiated with a tungsten-collimated high-dose-rate ^192^Ir beam.^16^

### Animals

All animal studies were approved by the Mayo Clinic Institutional Animal Care and Use Committee. Healthy FVB mice were used for Studies 1, 4, and 5, and C57BL/6 mice for Studies 2 and 3. In Study 6, GBM PDX tumors were established in female athymic nude mice by intracranial injection of GBM cells.^17^

### LC-MS/MS Analysis and Concentration Calculation

Harvested plasma and tissue samples were flash-frozen and stored at −80 °C until analysis. Drug concentrations were determined using LC-MS/MS assays. Levetiracetam, cefazolin, brigimadlin, and nedisertib were analyzed by Micromass Quattro Ultima mass spectrometer coupled with AQUITY UPLC system.^18,19^ GNE-317 and apitolisib were analyzed with a TSQ Quantum triple quadrupole mass spectrometer linked to an Agilent Technologies model 1200 HPLC system.^20^ Additional details for these LC-MS/MS assays are provided in Supplementary Methods. Brain and tumor concentrations were not corrected for the residual blood in the vasculature.

### Statistical Analysis

All ratios were log-transformed for analyses. A paired *t*-test was used to compare drug levels between 2 brain hemispheres. Comparisons between normal brains or tumor tissues treated with supratentorial irradiation were analyzed by a 2-sample *t*-test. *P* < .05 was considered statistically significant for all comparisons.

## Results

The effects of radiation on drug distribution into the CNS were evaluated in mice treated with ionizing radiation. To approximate the effects of conventional, fractionated radiation therapy used for the treatment of gliomas or brain metastases, 2 fractionation schedules were used in these studies. First, 30 Gy in 5 fractions is commonly used for fractionated stereotactic radiation surgery for the treatment of larger brain metastases,^21^ and 40 Gy in 10 fractions is a dosing regimen being used for the treatment of glioblastoma in clinical trials.^22^ For studies with relatively early endpoints, the right hemisphere was irradiated to enable the use of the contralateral, non-irradiated hemisphere as an internal control (Figure 1A). For studies with later timepoints, ‘whole-brain’ supratentorial irradiation was used to ensure the optimal health of the animals (Figure 1B). To develop a broader understanding of how radiation might influence drug distribution into the CNS, a set of 6 drugs with differing physico-chemical properties were selected for testing (Table 1), and drug distribution was examined at varying times after completion of radiation. For most studies, the test drugs were dosed at varying times after the last dose of radiation and plasma and tissue were subsequently collected (Figure 1C). For clarity in this section, the time interval from the last dose of radiation to plasma/tissue harvest is denoted in the text and graphs.

### Acute Effects of Radiation on Levetiracetam and Cefazolin Distribution into Normal Brain Tissues

Levetiracetam and cefazolin are given peri-operatively to brain tumor patients to prevent seizures and surgical site infection, respectively.^26,27^ Levetiracetam is highly brain penetrant,^23^ and differences in brain accumulation would be primarily dependent on changes in tissue perfusion. In contrast, cefazolin has very limited brain penetration,^24,25^ and any increase in drug distribution after radiation would reflect disruption of the BBB. Therefore, mice were irradiated with 30 Gy in 5 fractions on the right hemisphere, levetiracetam/cefazolin were co-administrated 15 minutes after the last dose of radiation, and then drug levels were measured 15 and 60 minutes later (30- and 75-minute groups, respectively; Figure 1C—Study 1). In this study, the levels of levetiracetam and cefazolin in the 2 hemispheres (non-irradiated vs. irradiated) were not significantly different in either the 30- or 75-minute groups after 30 Gy radiation treatment. The brain-to-plasma ratio of levetiracetam in non-irradiated and irradiated hemispheres was almost identical at both timepoints: 0.17 ± 0.01 versus 0.18 ± 0.01 (*P* = .51, *n* = 5) at 30 minutes, and 0.52 ± 0.03 vs. 0.52 ± 0.01 (*P* = .79, *n* = 5) at 75 minutes (Figure 2A, Table S1). The brain-to-plasma ratio of levetiracetam for both hemispheres was higher at 75 minutes when compared to that at 30 minutes consistent with sampling times before an equilibrium is reached between the brain and plasma compartments. In the same mice, the brain-to-plasma ratios of cefazolin in non-irradiated and irradiated hemispheres were remarkably similar: 0.008 ± 0.002 versus 0.008 ± 0.003 (*P* = .40, *n* = 5) at 30 minutes, and 0.007 ± 0.002 versus 0.007 ± 0.002 (*P* = .56, *n* = 5) at 75 minutes (Figure 2B, Table S2). Similar results were observed in the control group (0 Gy), where mice received sham hemi-brain radiation (Table S1, S2). No significant difference was seen between the non-irradiated and irradiated hemispheres at either time point.

### Effects of Radiation on Small Molecule PI3K/mTOR Inhibitor Distribution into Normal Brain

GNE-317 and apitolisib (GDC0980) have the same molecular targets (PI3K/mTOR) and similar physico-chemical properties, but markedly different BBB penetration related to differences in efflux transport.^20^ Healthy mice were treated with hemibrain irradiation (4 Gy × 10), and then both drugs were co-dosed at various intervals after the final radiation fraction (Figure 1C—Study 2). For early single (ie, acute) timepoints, eg, 3 and 65 hours, there was no significant difference in levels for either drug between the non-irradiated and irradiated hemispheres (Table S3, S4), resulting in similar brain-to-plasma ratios (GNE-317: 3 hours–0.83 ± 0.25 vs. 0.91 ± 0.33 (*P* = .14, *n* = 4), 65 hours—0.66 ± 0.15 vs. 0.70 ± 0.15 (*P* = .18, *n* = 5), respectively, Figure 3A; apitolisib: 3 hours—0.06 ± 0.03 vs. 0.06 ± 0.03 (*P* = .73, n = 4), 65 hours—0.04 ± 0.01 *vs.* 0.05 ± 0.02 (*P* = .19, *n* = 5); Figure 3B). Similarly, in the sham groups (0 Gy), there also was no significant difference in drug levels between 2 hemispheres (GNE-317–0.86 ± 0.24 vs. 0.98 ± 0.30 [*P* = .43, *n* = 3], apitolisib—0.06 ± 0.01 vs. 0.06 ± 0.01 [*P* = .73, *n* = 3]). The brain distribution of GNE-317 and apitolisib also was evaluated at longer timepoints after irradiation. At 2 weeks post-radiation, the relative brain exposures (brain-to-plasma ratios), at a single sampling timepoint, were similar between the non-irradiated and irradiated hemispheres for GNE-317 (0.55 ± 0.05 vs. 0.56 ± 0.05, respectively; *P* = .43, *n* = 5; Figure 3C, Table S5) and apitolisib (0.08 ± 0.02 vs. 0.10 ± 0.05, respectively; *P* = .30, *n* = 5; Figure 3D, Table S6). After 6 weeks, the brain-to-plasma ratios, again at a single sampling timepoint, were slightly higher in the non-irradiated hemispheres for GNE-317 (0.85 ± 0.15 vs. 0.72 ± 0.06; *P* = .03, *n* = 5) and apitolisib (0.31 ± 0.22 vs. 0.13 ± 0.06, respectively; *P* = .15, *n* = 5). Thus, a clinically used radiation regimen of 40 Gy in 10 fractions had no meaningful impact on the distribution of either PI3K/mTOR inhibitor at times ranging from hours to weeks after irradiation.

A supra-therapeutic dose of radiation also was evaluated in our model using the PI3K/mTOR inhibitors after a single high radiation dose. We delivered 40 Gy × 1 hemibrain radiation to healthy mice and then administered GNE-317 and apitolisib 17, 65, 113, and 161 hours post-radiation (Figure 1C—Study 3). As shown in Figure 3E and F, brain distribution of GNE-317 and apitolisib in paired non-irradiated and irradiated hemispheres are not significantly different (*P* > .05, *n* = 3), at all time points (Table S7, S8). Thus, drug delivery of both a brain-permeable drug (GNE-317) and a brain-impermeable drug (apitolisib) were not significantly affected even at a supra-therapeutic level of radiation.

### Acute Effects of Radiation on the Distribution of Radiosensitizer Nedisertib into Normal Brain

Acute effects of radiation also were examined with the radiosensitizer nedisertib, which has limited brain penetration.^18^ Ten minutes after radiation with 30 Gy in 5 fractions, FVB mice were dosed with nedisertib and drug exposure in both hemispheres was evaluated at 2 and 5 hours post-drug-dose (see Figure 1C—Study 4). Compared to the non-irradiated hemisphere, nedisertib showed a slightly higher, but not significant, brain-to-plasma ratio at both 2 hours (0.05 ± 0.01 vs. 0.07 ± 0.02, respectively; *P* = .06, *n* = 4) and 5 hours (0.07 ± 0.03 vs. 0.09 ± 0.03, respectively; *P* = .13, *n* = 4) post-dose in the irradiated hemispheres (Figure 4A, Table S9). These results suggest that radiation has subtle effects on the permeability of nedisertib across the BBB at an early time-point after radiation doses are used in fractionated radiosurgery.

### Acute and Delayed Effects of Radiation on Brain Distribution of a Potent MDM2 Inhibitor

Brigimadlin is a potent MDM2 inhibitor that is currently being studied in a clinical trial for newly diagnosed GBM patients (NCT05376800). The brain distribution of brigimadlin is very limited, in large part due to efflux transport at the BBB.^19^ Therefore, the brain distribution of this compound could be highly sensitive to alterations in BBB permeability, either due to changes in efflux transport tight junction integrity, or both. In this study, 2 different time points after hemi-brain irradiation (30 Gy in 5 fractions) were studied. Cohorts of animals were dosed with brigimadlin 15 minutes or 48 hours after completion of radiation, and samples were collected 24 hours later to provide evaluations of brain exposure 24 and 72 hours after the last radiation dose (see Figure 1C—Study 5). At the 24-hour post-radiation timepoint, brigimadlin brain-to-plasma ratios were similar between non-irradiated (0.02 ± 0.01) and irradiated (0.02 ± 0.01) hemispheres (*P* = .63, *n* = 5; Figure 4B, Table S10). Compared to the non-irradiated hemisphere 72 hours post-radiation, brigimadlin accumulated within the irradiated hemisphere at significantly higher levels (brain-to-plasma ratio of 0.01 ± 0.01 vs. 0.03 ± 0.01, respectively; *P* = .04, *n* = 5). Thus, similar to nedisertib, radiation had subtle, but statistically significant effects on the distribution of brigimadlin into the brain.

The impact of radiation on brigimadlin distribution also was assessed at extended timepoints after treatment. FVB mice were randomized into cohorts of 4–5 mice, were treated with sham radiation or 6 Gy × 5 supratentorial irradiation, and then followed various times after radiation. For relatively early timepoints, mice were dosed with brigimadlin 14 or 27 days after irradiation, tissue was collected 1 day later, and brain and plasma brigimadlin concentrations were compared in the sham and irradiated groups (see Figure 1C—Study 5). Compared to the non-irradiation cohort, the brain-to-plasma ratio of brigimadlin at the single timepoint in the irradiated group was similar 15 days after radiation (0.025 ± 0.006 vs. 0.032 ± 0.004, respectively; *P* = .08, *n* = 5; Figure 4C, Table S11). Also, by 28 days after irradiation, there was no difference in the brain-to-plasma ratio (0.029 ± 0.001 vs. 0.033 ± 0.011, respectively; *P* = .67, *n* = 5; Figure 4C). Similarly, at extended timepoints, the brain-to-plasma ratio for brigimadlin without and with radiation treatment were not significantly different (94 days—0.039 ± 0.003 vs. 0.033 ± 0.010 (*P* = .24, *n* = 4 or 5); 133 days—0.026 ± 0.002 vs. 0.027 ± 0.006 (*P* = .78, *n* = 5), 183 days—0.031 ± 0.012 vs. 0.028 ± 0.007 (*P* = .82, *n* = 4 or 5); Figure 4D, Table S12). These data suggest that fractionated radiation (30 Gy in 5 fractions) does not have long-lasting effects on the brain distribution of brigimadlin.

### Radiation Effects on Drug Delivery in Orthotopic Brain Tumor Models

The BBB is perturbed and functionally distinct within brain tumors as compared to normal brains.^28^ Therefore, the influence of radiation on the delivery of brigimadlin and nedisertib was evaluated in 2 orthotopic GBM PDXs—GBM108 and GBM10. Tumor-bearing mice were treated with supratentorial irradiation to 6 Gy × 5 (or sham treatment) and brigimadlin or nedisertib were dosed immediately before the last radiation dose (see Figure 1C—Study 6).

In GBM108, normal brain-to-plasma concentration ratios of brigimadlin were similar between sham (0 Gy) and radiation (6 Gy × 5) treated mice (0.05 ± 0.01 vs. 0.04 ± 0.01, respectively; *P* = .67, *n* = 4 or 5), (Figure 5A). Tumor-to-plasma ratios of brigimadlin were much higher than normal brain-to-plasma ratios at 24 hours post-dosing, but not significantly different between groups without or with irradiation treatment (0.69 ± 0.46 vs. 0.29 ± 0.13, respectively; *P* = .08, *n* = 4 or 5; Figure 5B). However, nedisertib had significantly different brain-to-plasma ratios between sham (0 Gy) and radiation (6 Gy × 5) groups (0.07 ± 0.01 vs. 0.05 ± 0.01, respectively; *P* = .04, *n* = 4 or 5; Figure 5C) at 2 hours post-dosing, but identical tumor-to-plasma ratios (0.11 ± 0.03 vs. 0.11 ± 0.02, respectively; *P* = .96, *n* = 4 or 5; Figure 5D). Both brigimadlin and nedisertib had an increased distribution into intracranial GBM108 tumors when compared to the corresponding normal brain (Table S13, S14). This difference can be attributed to a leakier blood tumor barrier (BTB) in tumor regions than an intact BBB in normal brain tissues.^28^

The delivery of brigimadlin into GBM10 tumors also was evaluated using the same radiation and drug dosing schedule. The distribution of brigimadlin between 0 Gy and 6 Gy × 5 treatment groups was highly similar in both normal brain (0.05 ± 0.01 vs. 0.05 ± 0.01, respectively; *P* = .88, *n* = 6 or 9; Figure 5E) and brain tumor (0.22 ± 0.06 vs. 0.23 ± 0.09, respectively; *P* = .93, *n* = 6 or 9; Figure 5F). Similar to GBM108, brigimadlin had a higher distribution into intracranial GBM10 tumors than in the adjacent normal brain (Table S15). Although the tumor distribution of brigimadlin in GBM108 models was variable, the mean tumor-to-plasma ratios of brigimadlin in the sham-treated groups were higher than that in GBM10 models (0.69 ± 0.46 in GBM108 vs. 0.22 ± 0.06 in GBM10), possibly due to the heterogeneity of the BTB in different PDXs or greater drug binding in the *MDM2*-amplified GBM108 PDX.

## Discussion

Treatment of primary and metastatic cancers affecting the CNS poses some of the most daunting therapeutic challenges in oncology. While radiation therapy is commonly used as part of routine care for many of these patients, cognitive injury and other neurologic sequelae limit the radiation intensity of the treatment regimens used. Systemically administered chemotherapies have the potential to augment the efficacy of radiation, but the vast majority of chemotherapeutic agents are excluded from the CNS by the activity of the BBB. Therefore, defining effective strategies to enhance drug delivery across the BBB could have major therapeutic benefits. There are multiple preclinical and clinical studies demonstrating the effects of radiation on the BBB based on measurement of biomarkers of BBB integrity,^12–14^ but there has not been a direct evaluation of how radiation affects delivery of therapeutic agents into brain tissue. In this study, the effect of radiation on brain distribution of 6 small-molecule drugs was measured in normal mouse brains and in orthotopic GBM PDXs. These compounds have various physicochemical properties and BBB efflux liabilities (Table 1), and their pharmacological effects are diverse, ranging from antibiotic to antitumor agents. Consistent with prior results, radiation did enhance drug uptake into the brain, but the effects were subtle, transient, and unlikely to be clinically meaningful.

There is extensive literature detailing the effects of therapeutic radiation on various components of the BBB.^2,9–11^ Therapeutic electromagnetic radiations (X-rays, gamma rays) are sparsely ionizing and interact with inner or outer shell electrons to transfer energy into matter through the liberation of high-energy electrons. Biologic effects of irradiation are mediated through the generation of hydroxyl free radicals when resulting high-energy electrons interact with water.^29^ These free radicals are short-lived and almost immediately chemically modify adjacent biological molecules, such as proteins, lipids, and nucleic acids. Acutely, this burst of free radicals triggers a highly orchestrated response to cellular and genomic stress with numerous signaling pathways transiently altered. However, with random distribution across innumerable molecules throughout the cell, these stochastic events have limited lasting biological impact on the cell with the exception of damage to genomic DNA. Inadequate repair of genomic DNA is a major contributor to genomic instability, promotes malignant transformation, and can cause cytotoxicity in both tumor and normal cells. The time scales for these biological events vary widely with acute intracellular signaling responses to radiation occurring within seconds to hours of exposure, while cell death occurs over days to weeks after radiation.^30^ Beyond direct cellular effects, the biologic sequelae of radiation exposure result in microenvironmental changes mediated by complex inflammatory responses.^31^ Within this biological context, various groups have reported biochemical changes within the BBB associated with reduced expression of tight junction proteins (eg occludin and claudin-5), changes in expression or activity of efflux transporters active within the BBB, senescence of brain endothelial cells, pericytes, and astrocytes that collectively maintain the BBB, and inflammatory reactions within the brain parenchyma.^2,12,31–33^ With the clinically relevant radiation doses used in the present study, radiation likely evoked many of these reported subtle physiologic effects on the BBB; however, these effects did not translate into major changes in drug distribution into normal brain or brain tumors for the 6 drugs measured.

The effects of radiation on the BBB have been most extensively studied at the level of the physical integrity of junctions between brain capillary endothelial cells. These studies uniformly rely on measuring the accumulation of biomarkers in brain tissue (eg, TexasRed, FITC-dextran,^14^C-sucrose,^14^C-alpha-aminoisobuturic acid, magnetic resonance imaging (MRI) or computed tomography contrast agents) that all are dependent on paracellular diffusion across disrupted physical junctions between brain capillary endothelial cells.^14^ For example, a single fraction of 15.5 Gy whole brain radiation in mice resulted in an approximately 2 to 3-fold increase in brain distribution for several different tracers that peaked 12 hours after irradiation.^12^ In a more protracted mouse study of fractionated radiation (2 Gy × 20 fractions over 4 weeks), an approximately 2-fold increase in BBB permeability to FITC-dextran molecules was detected between 90 and 180 days after completion of radiation in association with gliosis but retained tight-junction integrity.^11^ Key takeaways from these 2 examples are as follows: (1) the biomarkers used have minimal distribution across an unperturbed BBB, (2) the detection methodologies used are highly sensitive, and (3) while the relative change in distribution may be double or triple, the change in absolute concentrations for the measured biomarkers are small. Importantly, the intensity of the radiation regimens used in these examples are clinically relevant and comparable to those used in our study (30 Gy in 5 fractions, 40 Gy in 10 fractions). Thus, our results reporting relatively subtle, often not statistically significant, changes in drug distribution in the brain following fractionated radiation are consistent with other previous preclinical quantitative studies.

The majority of clinical studies evaluating the effects of radiation on the BBB have relied on MRI. MRI is routinely used to image brain tumor patients before and after administration of paramagnetic gadolinium contrast. These contrast agents are hydrophilic and, similar to the biomarkers discussed above, only accumulate in regions of brain or brain tumors with a physically disrupted BBB. The MR signals on T1-weighted sequences are exquisitely sensitive to changes in gadolinium concentrations, and this provides a sensitive and accurate method to measure even subtle changes in BBB integrity. Within this framework, numerous clinical studies have demonstrated changes in contrast accumulation in brain and brain tumors following irradiation. For example, Cao et al. quantitatively assessed the effects of RT on the BBB permeability in glioma patients using MRI-measured changes in contrast accumulation to calculate a “contrast uptake index.”^13^ They demonstrated an increased uptake index in regions that originally lacked contrast enhancement (non-enhanced tumor) within a week after completing radiation with a peak at 3 weeks and resolution after 1 month. Interestingly, in normal brain regions, no differences were observed in the contrast uptake index. In another example, Teng et al. evaluated radiationinduced changes in contrast accumulation in patients with treated brain metastases.^34^ They found the kinetics of contrast accumulation, measured as a transfer constant (K^trans^), was increased after radiation in the low-permeable metastatic lesions but not in high-permeable lesions. These examples motivated our analyses of changes in drug distribution in orthotopic PDX tumors following radiation. In our analyses, we only analyzed drug distribution immediately after the completion of fractionated radiation, since tumor shrinkage after radiation and/or regrowth of tumors would complicate our analysis in this animal model. Nonetheless, the lack of appreciable changes in drug distribution for 2 different PDX models at the end of a clinically relevant dosing regimen is consistent with a lack of profound changes in BBB integrity following irradiation observed in multiple human imaging studies.

Several limitations of this study are worth highlighting. Some of the experiments had noticeable variability in the measured plasma levels. Especially for cefazolin, with a half-life of approximately 20 minutes (internal data), the rapidly changing drug levels in individual animals introduce greater inherent variability with single time point sampling. A more rigorous, but labor-intensive approach, would be to sample mice at multiple times after drug dosing to build a concentration-time profile and then compare differences in the area-under-the-curve measure of drug exposure. In the studies with GNE-317/apitolisib, the plasma concentrations varied quite significantly in several animals. These studies were performed several years ago, and we attribute this variability to the technical inexperience of the individuals performing oral gavage dosing. However, with a paired analysis of drug levels in irradiated and non-irradiated hemispheres from in the same animal, this variability provides an informal assessment of how variable plasma concentrations, which provide the driving force for brain partitioning, affect drug accumulation following radiation. Finally, the drugs tested represent a small fraction of potentially clinically relevant agents used to treat malignancies affecting the CNS. Despite these caveats, the physiology of the BBB is highly similar between mouse and human, and the lack of meaningful effects of radiation on drug distribution demonstrated in these mouse models are likely highly relevant for patients with brain tumors.

There is considerable clinical interest in developing therapeutic strategies to disrupt the BBB in order to improve the delivery of novel therapeutics into brain tumors. Hyperosmolar therapy or focused ultrasound combined with microbubbles are 2 well-established techniques that disrupt the tight junctions between adjacent brain capillary endothelial cells.^35,36^ As compared to the relatively subtle changes in contrast enhancement following radiation, both of these BBB-disruption techniques cause readily apparent changes in gadolinium contrast enhancement and corresponding increases in drug accumulation within brain tumor tissue.^36–38^ Conceptually, the orders of magnitude difference in physical BBB disruption can be explained partially by the amount of energy being imparted to the brain vasculature. In the case of focused ultrasound, disruption of the tight junctions between endothelial cells relies on establishing harmonic oscillations of intravascular microbubbles that physically distort the capillary walls. As a qualitative understanding of the amount of energy being imparted with this technique, the intensity of the ultrasound is carefully monitored using MR thermography to limit the risk of heat generation within the sonicated brain. In contrast, based on the specific heat capacity of water, over 4000 Gy is required to increase the temperature of water by 1 °C. Similarly, in contrast with the rapid and easily apparent increase in contrast enhancement seen with either focused ultrasound or osmotic BBB disruption, high-dose fractionated radiation does not result in clinically appreciable changes in contrast enhancement within regions of the normal brain. Thus, the data presented in this manuscript that demonstrate extremely limited impacts of radiation on the gross disruption of the BBB, as measured by changes in drug accumulation, are consistent with the sparsely ionizing, stochastic nature of therapeutic radiation.

## Conclusions

Numerous published studies seemingly provide a rationale for careful sequencing of radiation and chemotherapy treatments to capitalize on the potential for radiation to disrupt the BBB and thereby improve drug delivery. This report is the first-ever study that directly tested this hypothesis through the quantitative measurement of drug concentrations in the brain and brain tumors at various timepoints after irradiation. Our results reveal that when the mouse brain was treated with clinically relevant radiation schedules, no therapeutically meaningful enhancement of drug delivery was observed. These preclinical data are consistent with the absence of compelling data that conventionally fractionated cranial irradiation causes clinically obvious changes in contrast enhancement within normal brain or brain tumors. We conclude from the current study that the common belief that radiation can meaningfully improve drug exposure in the brain needs to be reconsidered.

## Supplementary Material

Supplementary Tables

Supplementary Methods

Supplementary material is available online at *Neuro-Oncology* (https://academic.oup.com/neuro-oncology).

## Figures and Tables

**Figure 1.**
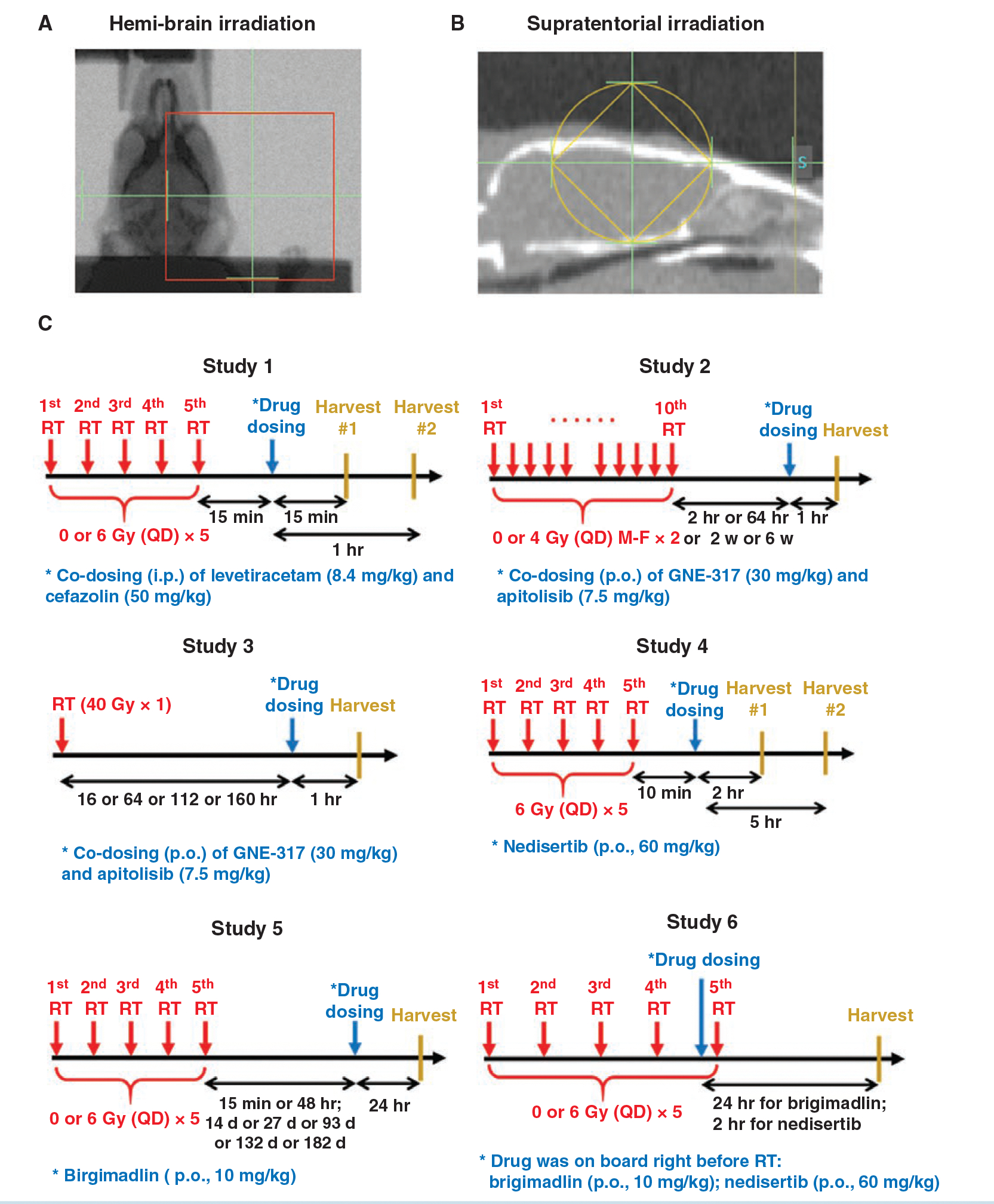
Radiation treatment setup and study design. Mice were treated with hemi-brain irradiation to the right side of the brain with a posterior to anterior beam (A) or supratentorial irradiation with opposed lateral beams (B). Various radiation and drug dosing schedules were studied as detailed in (C).

**Figure 2.**
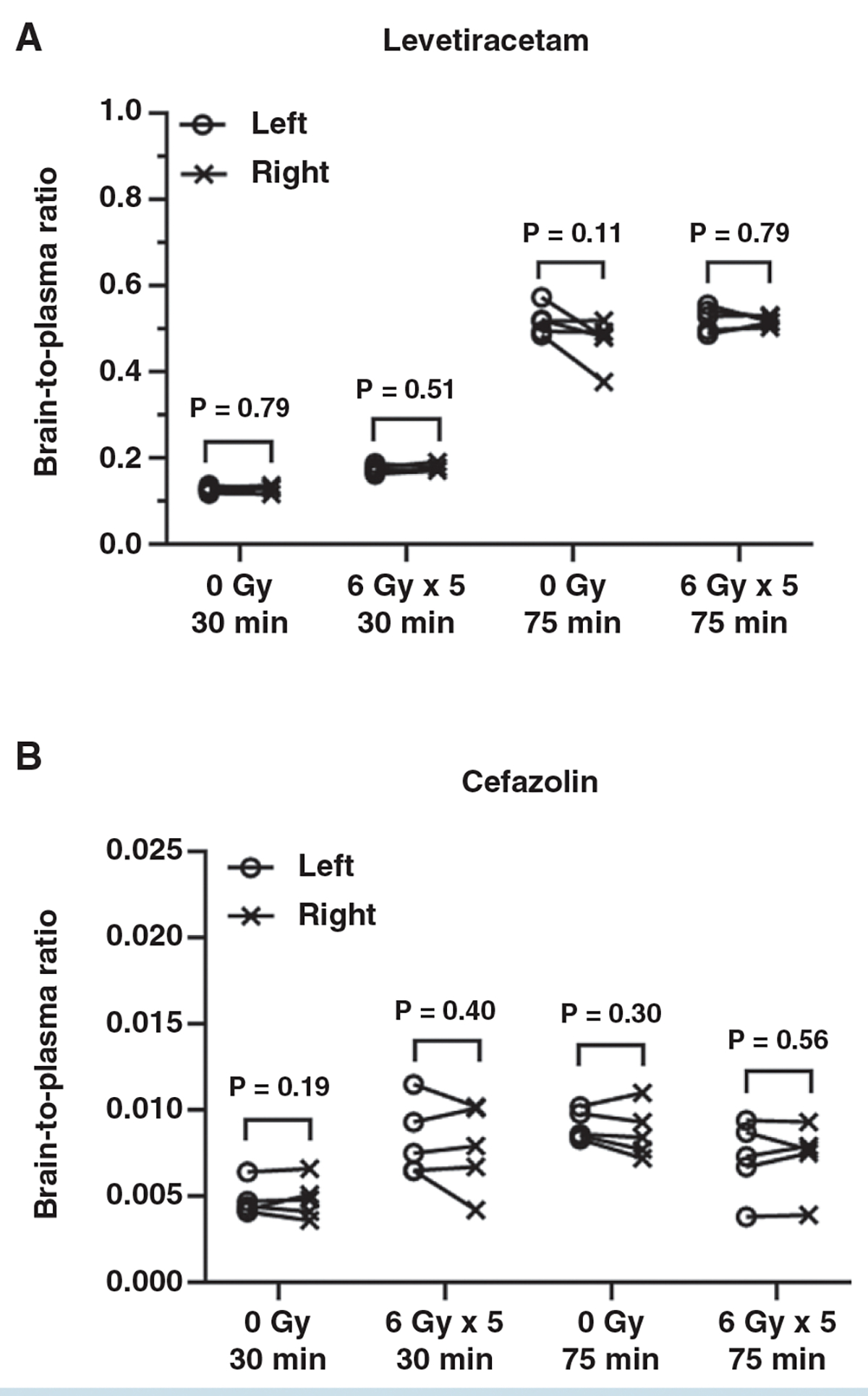
Acute effects of radiation on the distribution of levetiracetam and cefazolin in normal brain. Mice were treated with sham (0 Gy) or hemi-brain irradiation to the right hemisphere with 6 Gy per fraction, once daily for 5 days. Levetiracetam (8.4 mg/kg)) and cefazolin (50 mg/kg) were co-administrated 15 minutes after the last radiation treatment. Drug concentrations of levetiracetam (A) and cefazolin (B) were measured in paired non-irradiated and irradiated hemispheres at 30 minutes or 75 minutes post radiation. The two hemispheres from the same mouse are connected by a line, and were compared using a paired t-test.

**Figure 3.**
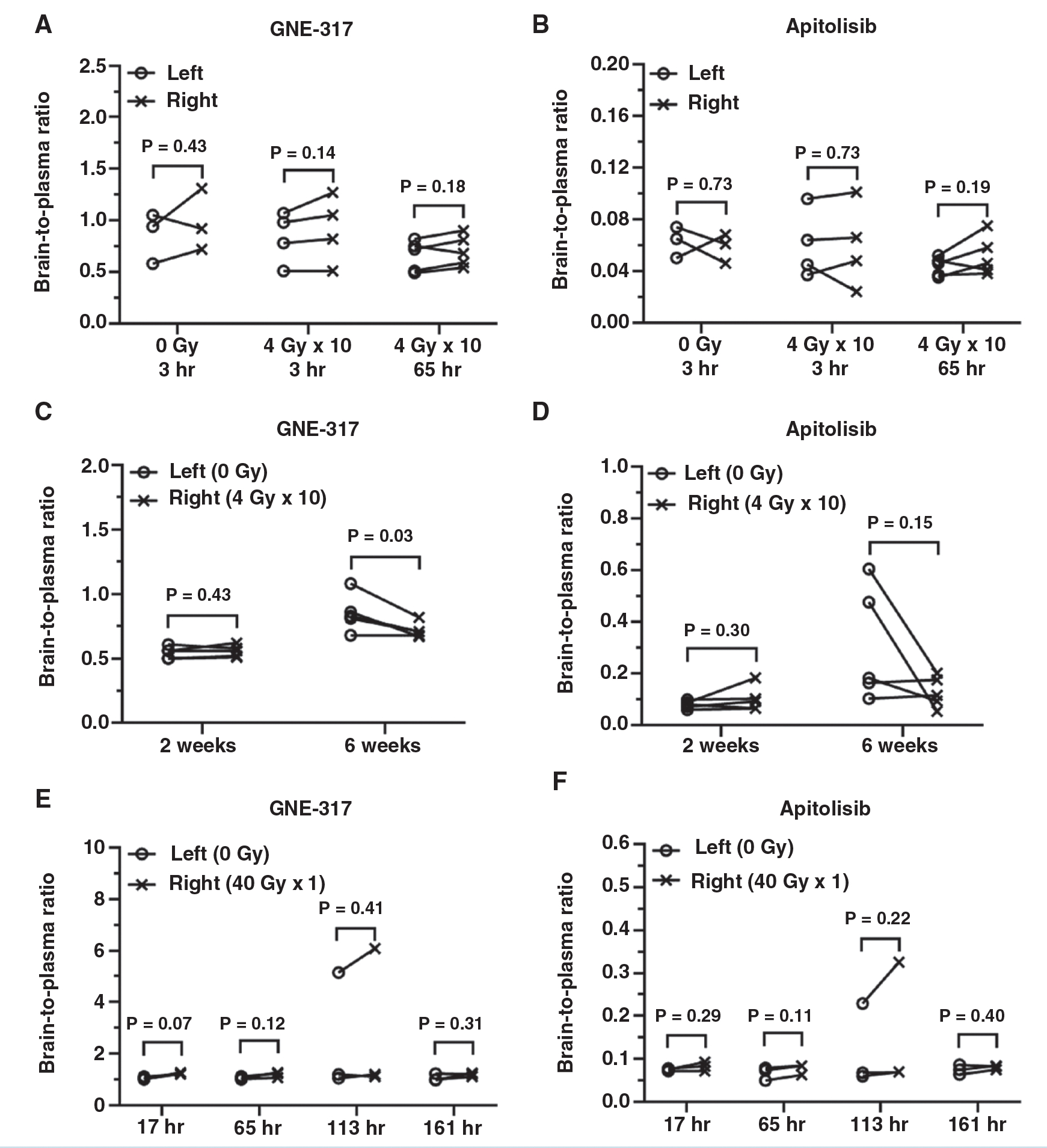
Effects of radiation on the distribution of PI3K/mTOR inhibitors in normal brain. Mice were treated with sham (0 Gy) or hemi-brain irradiation to the right hemisphere with 4 Gy once daily, Monday-Friday for 10 days (A-D) or 40 Gy in a single dose (E-F). GNE-317 (30 mg/kg) and apitolisib (7.5 mg/kg) were orally co-administrated at 2 and 64 hours (A, B) or 2 and 6 weeks (C, D) after the last radiation treatment. GNE-317 and apitolisib also were co-administrated at 16, 64, 112, or 160 hours after a single 40 Gy treatment (E, F). Drug concentrations of GNE-317 and apitolisib were measured in paired non-irradiated and irradiated hemispheres in mice. The two hemispheres from the same mouse are connected by a line and the data were compared using a paired t-test.

**Figure 4.**
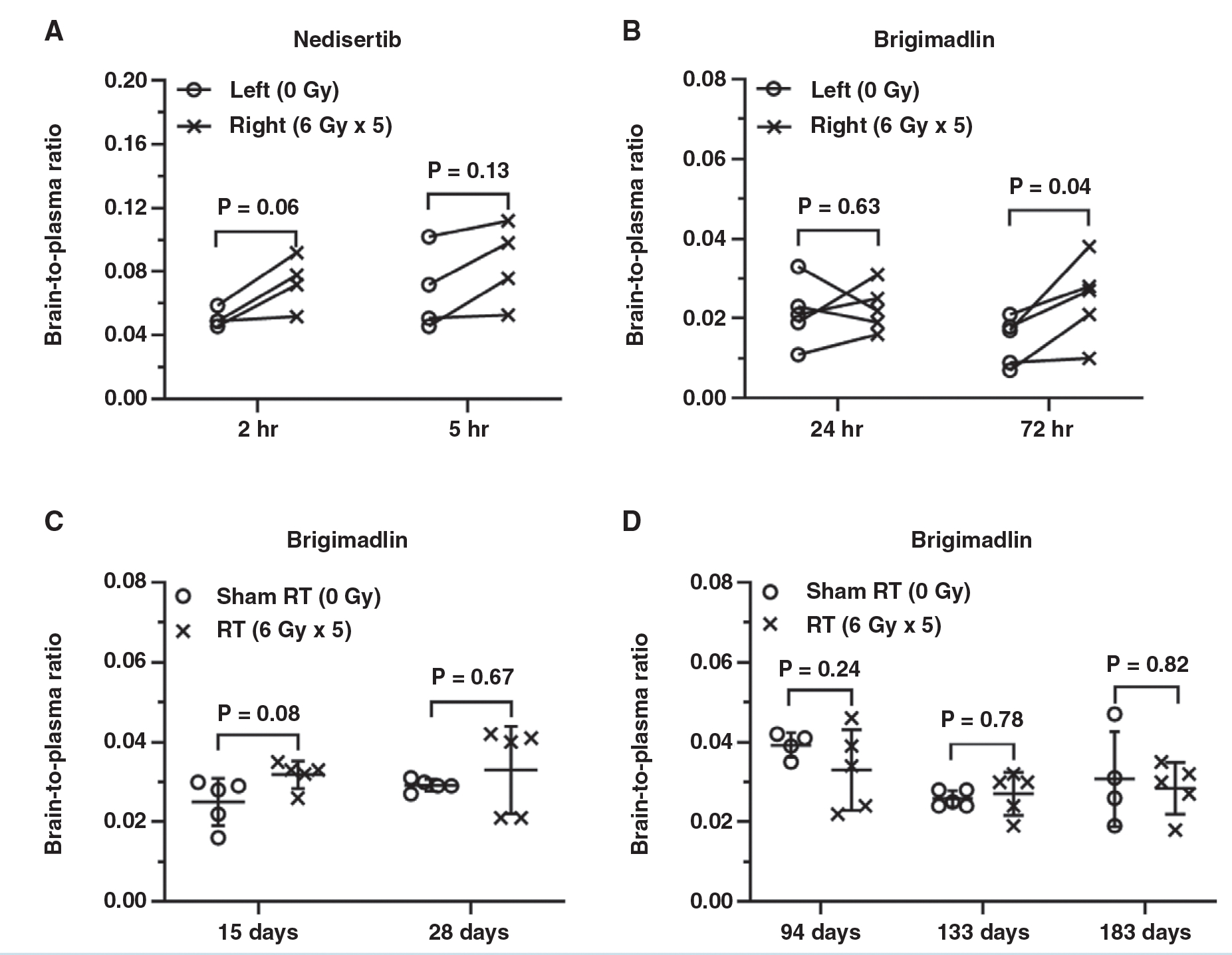
Effects of radiation on the distribution of nedisertib and brigimadlin in normal brain. Mice were treated with hemi-brain (A, B) or supratentorial (C, D) irradiation with 6 Gy once daily for 5 days. The brain and plasma concentrations of nedisertib (60 mg/kg) was measured 2 and 5 hours post oral dosing (A). Brigimadlin (10 mg/kg) concentrations in brain and plasma were measured at 24 or 72 hours (B), 15 or 28 days (C), and 94, 133 or 183 days (D) after the last radiation treatment. The two hemispheres from the same mouse are connected by a line (A and B only), and were compared using a paired t-test. Groups in the panels (C) and (D) were compared using a two-sample t-test.

**Figure 5.**
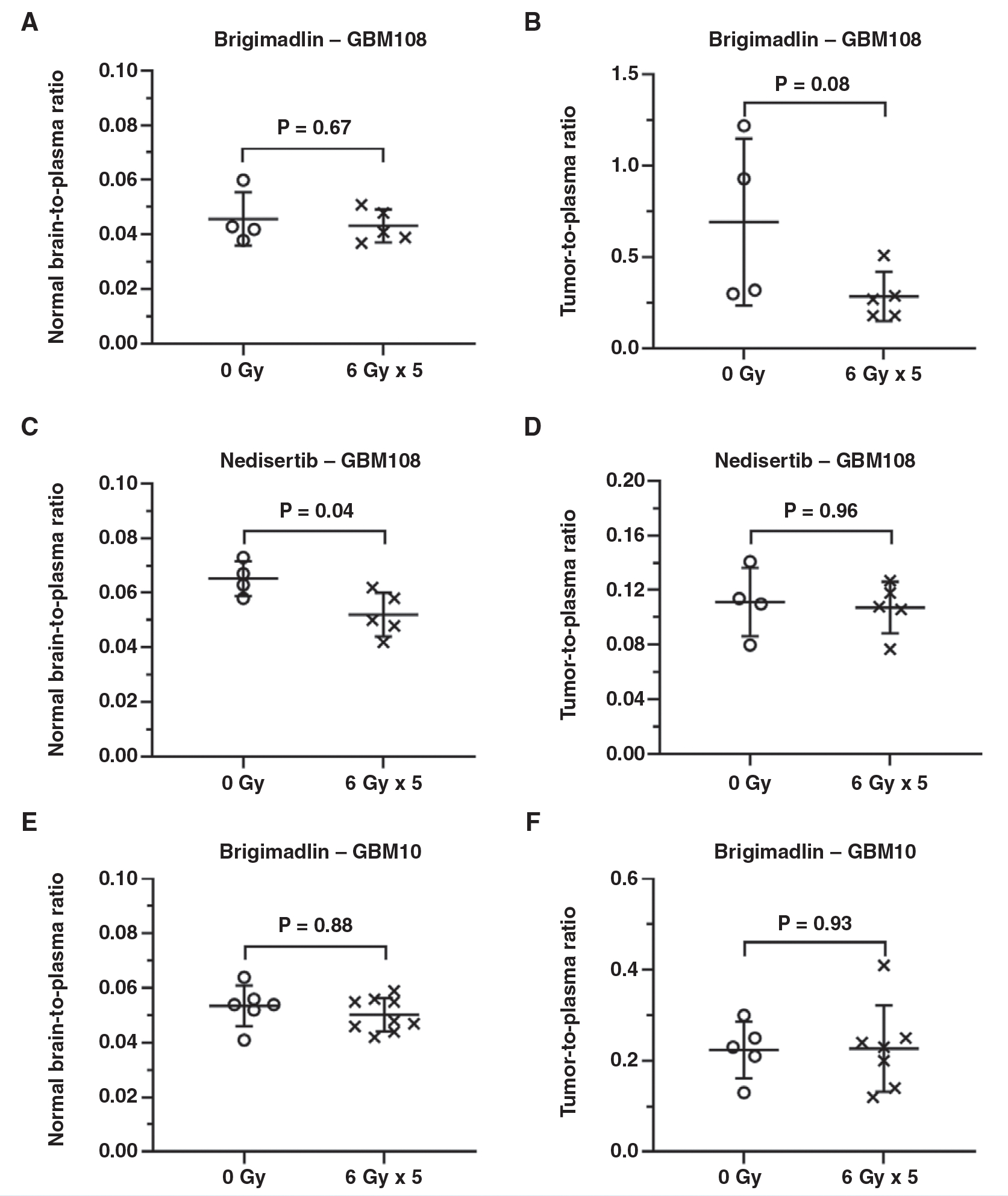
Effect of radiation on the BBB and BTB in orthotopic PDX brain tumor models. Mice with established GBM108 eGFP/fLuc2 (A-D) or GBM10 (E-F) intracranial tumors were treated with sham (0 Gy) or 6 Gy radiation once daily for 5 days. Brigimadlin (10 mg/kg) or nedisertib (60 mg/kg) were dosed orally right before the last radiation treatment. Mouse plasma, intracranial tumors, and surrounding normal brains were harvested at 24 hours (brigimadlin) or 2 hours (nedisertib) post drug dosing. The normal brain-to-plasma concentration ratios are shown in (A), (C), (E), and tumor-to-plasma ratios are shown in (B), (D), and (F). Comparisons were made using a two-sample t-test.

**Table 1.** Physico-Chemical Properties, Brain Distribution and Efflux Transporter Liability of Tested Compounds in This Study

Drug name	Chemical structure	MW (g/mol)	LogP	Kp_,brain_	BBB penetration	Efflux liability
Levetiracetam	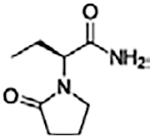	170.21	−0.6	0.50 ^[a]^	favorable	Not substrate of either P-gp or Bcrp ^[a]^
Cefazolin	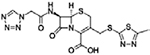	454.5	−0.58	0.06 ^[b]^	limited	Likely efflux by organic anion transporters ^[c]^
Nedisertib	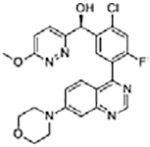	481.9	2.8*	0.15 ^[d]^	limited	Substrate of P-gp and Bcrp ^[d]^
Brigimadlin	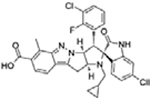	591.5	3.4*	0.009 ^[e]^	limited	Substrate of P-gp ^[e]^
Apitolisib	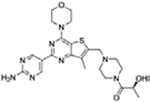	498.6	0.2*	0.1 ^[f]^	limited	Substrate of P-gp and Bcrp. P-gp is dominant ^[f]^
GNE-317	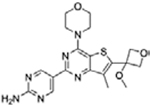	414.5	0.5*	0.81 ^[f]^	favorable	Not substrate of either P-gp or Bcrp ^[f]^

* means calculated Log P based on the XLogP3-AA, a method that uses atom-additive calculations to determine a molecule’s log P References:

[a] ,^23^

[b] ,^24^

[c] ,^25^

[d] ,^18^

[e] ,^19^

[f] .^20^.

## Data Availability

The summary data generated in this study are available within the article and most of the raw data are provided in the supplemental tables. Additional raw data will be provided upon request.

## Key Points

1. Radiation subtly enhances drug delivery across the BBB at early timepoints.
2. Radiation had no impact on drug distribution 1 week or later after treatment.
3. Changes in drug delivery to the brain caused by radiation are unlikely to be clinically relevant.

## Importance of the Study

Therapeutic radiation results in subtle disruption of BBB integrity measured by magnetic resonance imaging, and these data have been used to argue that radiation “opens the BBB.” However, no prior studies have evaluated the impact of radiation on drug distribution across the BBB. In this study, the effects of radiation on drug distribution into the brain and into brain tumors were studied. While subtle effects were observed within days of radiation, the effects on drug distribution were small, transient, and unlikely to be clinically meaningful. These data highlight the importance of re-evaluating the often-cited statement that radiation “disrupts the BBB” in the context of attempting to enhance drug delivery.
