# Supplementary material for: The impact of therapeutic radiation on drug distribution across the blood-brain barrier in normal mouse brain and orthotopic glioblastoma tumors
Source: Neuro Oncol. 2025 Mar 31;27(9):2250–61. doi: 10.1093/neuonc/noaf093 (PMC12403047; doi:10.1093/neuonc/noaf093)
Supplement: noaf093_suppl_Supplementary_Material [file noaf093_suppl_supplementary_material.docx]

**Supplemental Materials and Methods**

**Drug formulations and dosing**

Six drugs with different BBB permeabilities were used in this study. The anti-epileptic levetiracetam (Hikma Farmaceutica (Portugal) S.A.) and the antibiotic cefazolin (Hikma Farmaceutica (Portugal) S.A.) were co-administered to mice intraperitoneally. A fresh formulation was prepared on the day of dosing by dissolving levetiracetam and cefazolin sodium in saline. Cefazolin was protected from light until dosing and during bioanalysis. Brigimadlin (BI-907828, Boehringer Ingelheim, Ingelheim/Rhein, Germany), an MDM2-p53 antagonist, was formulated in 0.5% Natrosol^TM^ (Ashland^TM^, Wilmington, DE. 5.25 μL/mg 1M NaOH) for oral administration *via* oral gavage. Nedisertib (peposertib, M3814; Cancer Therapy Evaluation Program, NCI, Bethesda, MD), a DNA-dependent protein kinase inhibitor, was suspended in 0.25% hydroxypropyl methylcellulose and 0.25% Tween 20 in sodium citrate buffer (500 mM, pH 2.5) for oral administration. GNE-317 and apitolisib (GDC-0980, Genentech Inc., South San Francisco, CA), structurally related PI3K/mTOR inhibitors, were suspended in 0.5% methyl cellulose with 0.2% Tween 80 for co-administration orally.

**Study design**

The impact of radiation exposure on drug distribution into the brain was investigated in a series of experiments. To evaluate the potential temporal effects on the BBB, individual study designs examined acute (hours to days), subacute (weeks), and chronic (months) effects of radiation on CNS distribution of drugs with various innate BBB permeabilities (Figure 1).

- **Study 1**: Acute effects of radiation on the BBB permeability of clinical marker molecules (levetiracetam and cefazolin) was evaluated in healthy FVB mice. Mice (n=10 per group) were treated with sham or hemi-brain radiation to 30 Gy in 5 fractions. Levetiracetam (8.4 mg/kg) and cefazolin (50 mg/kg) were co-dosed 15 minutes after the last radiation treatment by intraperitoneal (i.p.) injection. Half of the mice (n=5) in each group were euthanized 15 minutes post drug dosing, and the other half were euthanized at 1 hour post-dose. Supratentorial brain (irradiated and non-irradiated hemispheres) and plasma were harvested from each mouse and flash frozen.
- **Study 2**: Acute and subacute effects of radiation on BBB permeability of two structurally related PI3K/mTOR inhibitors that differ significantly in their efflux liability (GNE-317 (brain penetrant) and apitolisib (brain impenetrant)) were evaluated in healthy C57BL/6 mice. This study was performed before our stereotactic animal irradiation system was available and used an ^192^Ir high-dose afterloader and custom tungsten collimator to deliver hemi-brain radiation. Mice (n=4 or 5) were treated with 0 Gy or hemi-brain irradiation (40 Gy in 10 fractions). GNE-317 (30 mg/kg) and apitolisib (7.5 mg/kg) were co-dosed 2 hours, 64 hours, 2 weeks, or 6 weeks after the last radiation treatment. Treated mice were euthanized at 1 hour post drug dosing. Plasma and both hemispheres from each mouse were harvested and flash frozen.
- **Study 3**: Acute effects of single high dose of radiation on the BBB permeability of GNE-317 and apitolisib were evaluated in healthy C57BL/6 mice. Mice (n=2 or 3) were treated with a supratherapeutic, single 40 Gy fraction of hemi-brain radiation irradiation. GNE-317 (30 mg/kg) and apitolisib (7.5 mg/kg) were orally co-dosed 16 hours, 64 hours, 112 hours, or 160 hours after radiation. Mice were euthanized at 1 hour post drug dosing. Plasma and both hemispheres from each mouse were harvested and flash frozen.
- **Study 4**: Acute effect of radiation on the BBB permeability of nedisertib, with limited brain penetration, was evaluated in FVB mice. Mice (n=10 per group) were treated with hemi-brain irradiation 30 Gy in 5 fractions. Nedisertib (60 mg/kg) was orally dosed 10 minutes after the last radiation treatment. Half of the mice (n=5) were euthanized at 2 hours post drug dosing, and the other half (n=5) were euthanized at 5 hours. Plasma and both hemispheres from each mouse were harvested and flash frozen.
- **Study 5**: Acute, subacute, and chronic effect of radiation on the BBB permeability of a brain impenetrable MDM2 inhibitor (brigimadlin) was evaluated in FVB mice. In the acute effect study, mice (n=5 per group) were treated with hemi-brain irradiation to 30 Gy in 5 fractions, and brigimadlin (10 mg/kg) was orally dosed 15 minutes or 48 hours after the last radiation dose. In this study, the unirradiated hemisphere served as an internal control. To examine effects of sub-acute (weeks) or chronic (months) radiation exposure, mice were treated with opposed lateral radiation to the supratentorial brain to prevent irradiation of the oral cavity and the associated acute toxicity. Drug exposure in brain and plasma were compared to sham irradiated mice. The same radiation regimen of 30 Gy in 5 fractions was used and brigimadlin (10 mg/kg) was orally dosed 14 days, 27 days, 93 days, 132 days, or 182 days after the last radiation dose (n=4 or 5 mice for each treatment). In both acute and delayed effect studies, treated mice were euthanized at 24 hours post drug dosing. Plasma and brain (irradiated and non-irradiated hemispheres in acute group, irradiated supratentorial brain in the delayed group) from each mouse were harvested and flash frozen.
- **Study 6**: Acute effects of radiation on the BBB permeability of brigimadlin and nedisertib in PDX brain tumor models were evaluated in GBM108-eGFPfLuc2 and GBM10 orthotopic xenografts. Intracranial tumor-bearing mice were treated with sham or supratentorial irradiation (30 Gy in 5 fractions). Brigimadlin (10 mg/kg) or nedisertib (60 mg/kg) were orally dosed immediately before the last radiation dose. Brigimadlin treated mice were euthanized at 24 hours post drug dosing, and nedisertib treated mice were euthanized at 2 hours post drug dosing, with plasma and brains were collected from each mouse. Intracranial tumor and contralateral normal brain were dissected from each animal under white-light (GBM10) or GFP-guidance (GBM108), and samples were flash frozen.

**Mouse radiation setup**

Radiation was delivered under general anesthesia with a mixture of 2.5% vaporized isoflurane and pure oxygen and immobilized using a stereotactic bite block. Irradiation for Studies 1-3 and 6 were performed using the X-RAD SmART irradiator (Precision X-ray Inc., Madison, CT) with integrated cone beam computed tomography (CBCT). Radiation was generated at 225 kVp, 20 mA with a 0.3 mm Cu filter. CBCT was used to accurately position the first animal treated for the day, and the same CBCT-based coordinates were used to treat all subsequent animals on that day. The intermouse positioning reproducibility of this method is on the order of 1 mm.^1^ For hemi-brain irradiation, a single posterior-anterior beam was delivered using a 20 mm square collimator, with the lateral edge of the beam setup along the midline of the head to deliver radiation only to the right side of the brain. For supratentorial irradiation, a 10 mm circular beam was delivered via opposed lateral beams. The SmART-ATP (Scientific Solutions B.V.) software package was used to determine the dose rate of this setup. Before the SmART irradiator was available at our institution, mice in Studies 2 and 3 were irradiated with a tungsten-collimated high-dose-rate ^192^Ir beam as described previously.^2^

**Animals**

All animal studies were approved by the Mayo Clinic Institutional Animal Care and Use Committee. Healthy FVB mice (Charles River) were used for Studies 1, 4, 5 and C57BL/6 mice (Harlan) were used for Studies 2 and 3. In Study 6, GBM PDX tumors (GBM108 and GBM10) were established in female athymic nude mice by intracranial injection of 3 × 10^5^ GBM cells suspended in PBS as previously described.^3^ To enable accurate tumor dissection, GBM108 cells were transduced with a modified pGIPZ lentiviral vector encoding a fusion of firefly luciferase (Luc2) with enhanced green fluorescent protein (eGFP). These intracranial GBM PDX models were used for experiments at 10-14 days after tumor implantation.

**LC-MS/MS analysis and concentration calculation**

Harvested plasma and tissue samples were flash frozen and stored at -80°C until analysis. Brain and tumor samples were prepared by adding 2X or 3X (w/v) 5% bovine serum albumin solution for homogenization using a tissue homogenizer. Drug concentrations in the above matrices were determined using LC-MS/MS assays as reported previously.^4–6^ Briefly, levetiracetam, cefazolin, brigimadlin, and nedisertib were analyzed by Micromass Quattro Ultima mass spectrometer coupled with AQUITY UPLC system. Levetiracetam was analyzed after protein precipitation by acetonitrile, with levetiracetam-d_6_ as the internal standard. Cefazolin was analyzed after liquid-liquid extraction by ethyl acetate at pH 1, and cefazolin-^13^C_2_, ^15^N was used as the internal standard. Brigimadlin was assessed after liquid-liquid extraction by ethyl acetate with navtemadlin used as the internal standard.^5^ Nedisertib was measured using liquid-liquid extraction by ethyl acetate with basic buffer (pH 11), and LMP-400 was used as internal standard for the analysis.^6^ GNE-317 and apitolisib were analyzed with a TSQ Quantum triple quadrupole mass spectrometer linked to an Agilent Technologies model 1200 HPLC system. Both GNE-317 and apitolisib were extracted from samples using liquid-liquid extraction by ethyl acetate, and AG1478 and dasatinib were used as the internal standards, respectively.^4^ The precision and accuracy of all assays has been previously reported.^4–6^

**References**

1. Walb MC, Carlson BL, Sarkaria JN, Tryggestad EJ. Quantifying the setup uncertainty of a stereotactic murine micro-image guided radiation therapy system. *Br J Radiol*. 2019;92(1095):20180487.

2. Grams MP, Wilson ZC, Sio TT, et al. Design and characterization of an economical (192)Ir hemi-brain small animal irradiator. *Int J Radiat Biol*. 2014;90(10):936-942.

3. Carlson BL, Pokorny JL, Schroeder MA, Sarkaria JN. Establishment, Maintenance, and In Vitro and In Vivo Applications of Primary Human Glioblastoma Multiforme (GBM) Xenograft Models for Translational Biology Studies and Drug Discovery. *Curr Protoc Pharmacol*. 2011;52(1):14.16.1-14.16.23.

4. Becker CM, Oberoi RK, McFarren SJ, et al. Decreased affinity for efflux transporters increases brain penetrance and molecular targeting of a PI3K/mTOR inhibitor in a mouse model of glioblastoma. *Neuro Oncol*. 2015;17(9):1210-1219.

5. Zhang W, Vaubel RA, Oh JH, et al. Delivery versus Potency in Treating Brain Tumors: BI-907828, a MDM2-p53 Antagonist with Limited BBB Penetration but Significant In Vivo Efficacy in Glioblastoma. *Mol Cancer Ther*. 2024;23(1):47-55.

6. Talele S, Zhang W, Oh JH, et al. Central Nervous System Delivery of the Catalytic Subunit of DNA-Dependent Protein Kinase Inhibitor Peposertib as Radiosensitizer for Brain Metastases. *J Pharmacol Exp Ther*. 2022;381(3):217-228.
