# Supplementary material for: The impact of therapeutic radiation on drug distribution across the blood-brain barrier in normal mouse brain and orthotopic glioblastoma tumors
Source: Neuro Oncol. 2025 Mar 31;27(9):2250–61. doi: 10.1093/neuonc/noaf093 (PMC12403047; doi:10.1093/neuonc/noaf093)
Supplement: noaf093_suppl_Supplementary_Tables [file noaf093_suppl_supplementary_tables.docx]

**Table S1.** Plasma and brain concentrations (uncorrected and corrected), and concentration ratios of levetiracetam 30 and 75 minutes after the last sham or radiation treatment (6 Gy x 5) in mice.

| **Levetiracetam (n=5)** | | | | | | | | | |
| --- | --- | --- | --- | --- | --- | --- | --- | --- | --- |
|  | **Plasma (ng/mL)** | **Without correction** | | | | **Corrected for brain vasculature (1.4% of brain weight)** | | | |
|  |  | **Concentration (ng/g brain)** | | **Brain-to-plasma ratio** | | **Concentration (ng/g brain)** | | **Brain-to-plasma ratio** | |
|  |  | **Non-irradiated** | **Irradiated** | **Non-irradiated** | **Irradiated** | **Non-irradiated** | **Irradiated** | **Non-irradiated** | **Irradiated** |
| Sham  (0 Gy) (30 min) | 13265 | 1560 | 1538 | 0.118 | 0.116 | 1374 | 1352 | 0.10 | 0.10 |
|  | 12554 | 1598 | 1641 | 0.127 | 0.131 | 1422 | 1465 | 0.11 | 0.12 |
|  | 11159 | 1451 | 1515 | 0.130 | 0.136 | 1295 | 1359 | 0.12 | 0.12 |
|  | 11015 | 1369 | 1425 | 0.124 | 0.129 | 1215 | 1271 | 0.11 | 0.12 |
|  | 8446 | 1146 | 1085 | 0.136 | 0.128 | 1028 | 967 | 0.12 | 0.11 |
| **Average** | **11288** | **1425** | **1441** | **0.127** | **0.128** | **1267** | **1283** | **0.11** | **0.11** |
| **SD** | **1849** | **180** | **213** | **0.007** | **0.007** | **155** | **190** | **0.01** | **0.01** |
| RT  (6 Gy x 5) (30 min) | 9816 | 1652 | 1669 | 0.168 | 0.170 | 1515 | 1532 | 0.15 | 0.16 |
|  | 10750 | 1741 | 1842 | 0.162 | 0.171 | 1590 | 1692 | 0.15 | 0.16 |
|  | 9453 | 1770 | 1685 | 0.187 | 0.178 | 1637 | 1552 | 0.17 | 0.16 |
|  | 9881 | 1761 | 1880 | 0.178 | 0.190 | 1622 | 1742 | 0.16 | 0.18 |
|  | 10597 | 1833 | 1820 | 0.173 | 0.172 | 1684 | 1671 | 0.16 | 0.16 |
| **Average** | **10100** | **1751** | **1779** | **0.174** | **0.176** | **1610** | **1638** | **0.16** | **0.16** |
| **SD** | **551** | **65** | **96** | **0.010** | **0.008** | **63** | **91** | **0.01** | **0.01** |
| Sham  (0 Gy) (75 min) | 6775 | 3882 | 3254 | 0.573 | 0.480 | 3787 | 3160 | 0.56 | 0.47 |
|  | 7433 | 3602 | 2794 | 0.485 | 0.376 | 3498 | 2690 | 0.47 | 0.36 |
|  | 6660 | 3462 | 3203 | 0.520 | 0.481 | 3369 | 3110 | 0.51 | 0.47 |
|  | 6997 | 3456 | 3452 | 0.494 | 0.493 | 3358 | 3354 | 0.48 | 0.48 |
|  | 6441 | 3334 | 3343 | 0.518 | 0.519 | 3244 | 3253 | 0.50 | 0.51 |
| **Average** | **6861** | **3547** | **3209** | **0.518** | **0.470** | **3451** | **3113** | **0.50** | **0.46** |
| **SD** | **378** | **210** | **251** | **0.034** | **0.055** | **208** | **254** | **0.03** | **0.05** |
| RT  (6 Gy x 5) (75 min) | 6568 | 3473 | 3489 | 0.529 | 0.531 | 3381 | 3397 | 0.51 | 0.52 |
|  | 6135 | 3046 | 3088 | 0.497 | 0.503 | 2961 | 3002 | 0.48 | 0.49 |
|  | 6630 | 3586 | 3492 | 0.541 | 0.527 | 3494 | 3399 | 0.53 | 0.51 |
|  | 5496 | 3052 | 2835 | 0.555 | 0.516 | 2975 | 2758 | 0.54 | 0.50 |
|  | 6827 | 3328 | 3508 | 0.487 | 0.514 | 3232 | 3412 | 0.47 | 0.50 |
| **Average** | **6331** | **3297** | **3282** | **0.522** | **0.518** | **3209** | **3194** | **0.51** | **0.50** |
| **SD** | **531** | **244** | **306** | **0.029** | **0.011** | **238** | **299** | **0.03** | **0.01** |

| **Cefazolin (n=5)** | | | | | | | | | |
| --- | --- | --- | --- | --- | --- | --- | --- | --- | --- |
|  | **Plasma (ng/mL)** | **Without correction** | | | | **Corrected for brain vasculature (1.4% of brain weight)** | | | |
|  |  | **Concentration (ng/g brain)** | | **Brain-to-plasma ratio** | | **Concentration (ng/g brain)** | | **Brain-to-plasma ratio** | |
|  |  | **Non-irradiated** | **Irradiated** | **Non-irradiated** | **Irradiated** | **Non-irradiated** | **Irradiated** | **Non-irradiated** | **Irradiated** |
| Sham (0 Gy) (30 min) | 200080 | 886 | 812 | 0.0044 | 0.0041 | -1915 | -1990 | NA | NA |
|  | 208120 | 972 | 998 | 0.0047 | 0.0048 | -1941 | -1916 | NA | NA |
|  | 190600 | 782 | 691 | 0.0041 | 0.0036 | -1886 | -1978 | NA | NA |
|  | 164520 | 686 | 835 | 0.0042 | 0.0051 | -1617 | -1468 | NA | NA |
|  | 88120 | 567 | 583 | 0.0064 | 0.0066 | -667 | -650 | NA | NA |
| **Average** | **170288** | **779** | **784** | **0.0048** | **0.0048** | **-1605** | **-1600** | **NA** | **NA** |
| **SD** | **48776** | **160** | **157** | **0.0010** | **0.0012** | **540** | **573** | **NA** | **NA** |
| RT (6 Gy x 5) (30 min) | 66040 | 616 | 666 | 0.0093 | 0.0101 | -309 | -259 | NA | NA |
|  | 118320 | 773 | 494 | 0.0065 | 0.0042 | -883 | -1162 | NA | NA |
|  | 85800 | 646 | 680 | 0.0075 | 0.0079 | -556 | -522 | NA | NA |
|  | 101360 | 659 | 680 | 0.0065 | 0.0067 | -760 | -739 | NA | NA |
|  | 83120 | 953 | 845 | 0.0115 | 0.0102 | -211 | -319 | NA | NA |
| **Average** | **90928** | **729** | **673** | **0.0083** | **0.0078** | **-544** | **-600** | **NA** | **NA** |
| **SD** | **19785** | **138** | **124** | **0.0021** | **0.0025** | **286** | **366** | **NA** | **NA** |
| Sham (0 Gy) (75 min) | 10006 | 102 | 110 | 0.0102 | 0.0110 | -38 | -30 | NA | NA |
|  | 13068 | 112 | 109 | 0.0086 | 0.0084 | -71 | -74 | NA | NA |
|  | 11966 | 102 | 92 | 0.0085 | 0.0077 | -66 | -76 | NA | NA |
|  | 11912 | 99 | 86 | 0.0083 | 0.0072 | -68 | -81 | NA | NA |
|  | 11512 | 112 | 107 | 0.0098 | 0.0093 | -49 | -54 | NA | NA |
| **Average** | **11693** | **105** | **101** | **0.0091** | **0.0087** | **-58** | **-63** | **NA** | **NA** |
| **SD** | **1106** | **6** | **11** | **0.0009** | **0.0015** | **14** | **21** | **NA** | **NA** |
| RT (6 Gy x 5) (75 min) | 9642 | 71 | 76 | 0.0073 | 0.0079 | -64 | -59 | NA | NA |
|  | 8818 | 83 | 82 | 0.0094 | 0.0093 | -40 | -42 | NA | NA |
|  | 19442 | 74 | 76 | 0.0038 | 0.0039 | -198 | -197 | NA | NA |
|  | 8872 | 77 | 68 | 0.0087 | 0.0077 | -47 | -56 | NA | NA |
|  | 11660 | 78 | 88 | 0.0067 | 0.0075 | -85 | -76 | NA | NA |
| **Average** | **11687** | **77** | **78** | **0.0072** | **0.0072** | **-87** | **-86** | **NA** | **NA** |
| **SD** | **4485** | **5** | **7** | **0.0022** | **0.0020** | **64** | **63** | **NA** | **NA** |

**Table S2.** Plasma and brain concentrations (uncorrected and corrected), and concentration ratios of cefazolin 30 and 75 minutes after the last sham or radiation treatment (6 Gy x 5) in mice.

NA: not applicable. With correction, the values of brain concentration were all negative.

**Table S3.** Plasma and brain concentrations (uncorrected and corrected), and concentration ratios of GNE-317 at 3 hours and 65 hours after the last sham or radiation treatment (4 Gy x 10) in mice.

| **GNE-317** | | | | | | | | | |
| --- | --- | --- | --- | --- | --- | --- | --- | --- | --- |
|  | **Plasma (ng/mL)** | **Without correction** | | | | **Corrected for brain vasculature (1.4% of brain weight)** | | | |
|  |  | **Concentration (ng/g brain)** | | **Brain-to-plasma ratio** | | **Concentration (ng/g brain)** | | **Brain-to-plasma ratio** | |
|  |  | **Non-irradiated** | **Irradiated** | **Non-irradiated** | **Irradiated** | **Non-irradiated** | **Irradiated** | **Non-irradiated** | **Irradiated** |
| Control (0 Gy) (3 hr) (n=3) | 2683 | 2815 | 2465 | 1.05 | 0.92 | 2777 | 2427 | 1.04 | 0.90 |
|  | 4574 | 2667 | 3283 | 0.58 | 0.72 | 2603 | 3219 | 0.57 | 0.70 |
|  | 968* | 908 | 1266 | 0.94 | 1.31 | 895 | 1253 | 0.92 | 1.29 |
| **Average** | **2741** | **2130** | **2338** | **0.86** | **0.98** | **2092** | **2300** | **0.84** | **0.97** |
| **SD** | **1804** | **1061** | **1014** | **0.24** | **0.30** | **1040** | **989** | **0.24** | **0.30** |
| RT (4 Gy x 10) (3 hr) (n=4) | 2178 | 2320 | 2767 | 1.07 | 1.27 | 2289 | 2737 | 1.05 | 1.26 |
|  | 4886 | 2498 | 2487 | 0.51 | 0.51 | 2429 | 2418 | 0.50 | 0.49 |
|  | 2358 | 2321 | 2482 | 0.98 | 1.05 | 2288 | 2449 | 0.97 | 1.04 |
|  | 1969 | 1531 | 1607 | 0.78 | 0.82 | 1504 | 1580 | 0.76 | 0.80 |
| **Average** | **2848** | **2168** | **2336** | **0.83** | **0.91** | **2127** | **2296** | **0.82** | **0.90** |
| **SD** | **1368** | **433** | **504** | **0.25** | **0.33** | **421** | **499** | **0.25** | **0.33** |
| RT (4 Gy x 10) (65 hr) (n=5) | 3577 | 2573 | 2889 | 0.72 | 0.81 | 2523 | 2839 | 0.71 | 0.79 |
|  | 3279 | 2452 | 2239 | 0.75 | 0.68 | 2406 | 2193 | 0.73 | 0.67 |
|  | 6143 | 3037 | 3289 | 0.49 | 0.54 | 2951 | 3203 | 0.48 | 0.52 |
|  | 3749 | 3092 | 3386 | 0.82 | 0.90 | 3039 | 3334 | 0.81 | 0.89 |
|  | 5972 | 3042 | 3521 | 0.51 | 0.59 | 2958 | 3437 | 0.50 | 0.58 |
| **Average** | **4544** | **2839** | **3065** | **0.66** | **0.70** | **2775** | **3001** | **0.65** | **0.69** |
| **SD** | **1393** | **302** | **518** | **0.15** | **0.15** | **289** | **505** | **0.15** | **0.15** |

* Low value may be caused by incomplete oral gavage.

**Table S4.** Plasma and brain concentrations (uncorrected and corrected), and concentration ratios of apitolisib at 3 hours and 65 hours after the last sham or radiation treatment (4 Gy x 10) in mice.

| **Apitolisib** | | | | | | | | | |
| --- | --- | --- | --- | --- | --- | --- | --- | --- | --- |
|  | **Plasma (ng/mL)** | **Without correction** | | | | **Corrected for brain vasculature (1.4% of brain weight)** | | | |
|  |  | **Concentration (ng/g brain)** | | **Brain-to-plasma ratio** | | **Concentration (ng/g brain)** | | **Brain-to-plasma ratio** | |
|  |  | **Non-irradiated** | **Irradiated** | **Non-irradiated** | **Irradiated** | **Non-irradiated** | **Irradiated** | **Non-irradiated** | **Irradiated** |
| Control (0 Gy) (3 hr) (n=3) | 3910 | 291 | 238 | 0.074 | 0.061 | 237 | 183 | 0.06 | 0.05 |
|  | 4490 | 291 | 208 | 0.065 | 0.046 | 228 | 145 | 0.05 | 0.03 |
|  | 517* | 26 | 35 | 0.050 | 0.068 | 18 | 28 | 0.04 | 0.05 |
| **Average** | **2972** | **203** | **160** | **0.063** | **0.058** | **161** | **119** | **0.05** | **0.04** |
| **SD** | **2146** | **153** | **110** | **0.012** | **0.011** | **124** | **81** | **0.01** | **0.01** |
| RT (4 Gy x 10) (3 hr) (n=4) | 5976 | 576 | 605 | 0.096 | 0.101 | 493 | 521 | 0.08 | 0.09 |
|  | 2377 | 107 | 56 | 0.045 | 0.024 | 74 | 23 | 0.03 | 0.01 |
|  | 1142 | 73 | 75 | 0.064 | 0.066 | 57 | 59 | 0.05 | 0.05 |
|  | 1384 | 51 | 67 | 0.037 | 0.048 | 31 | 48 | 0.02 | 0.03 |
| **Average** | **2720** | **202** | **201** | **0.061** | **0.060** | **164** | **163** | **0.05** | **0.05** |
| **SD** | **2236** | **251** | **270** | **0.026** | **0.033** | **220** | **240** | **0.03** | **0.03** |
| RT (4 Gy x 10) (65 hr) (n=5) | 2073 | 96 | 120 | 0.046 | 0.058 | 67 | 91 | 0.03 | 0.04 |
|  | 1075 | 52 | 44 | 0.048 | 0.041 | 37 | 29 | 0.03 | 0.03 |
|  | 2726 | 102 | 103 | 0.037 | 0.038 | 64 | 65 | 0.02 | 0.02 |
|  | 479 | 25 | 36 | 0.052 | 0.075 | 19 | 29 | 0.04 | 0.06 |
|  | 973 | 34 | 45 | 0.035 | 0.046 | 21 | 32 | 0.02 | 0.03 |
| **Average** | **1465** | **62** | **70** | **0.044** | **0.052** | **41** | **49** | **0.03** | **0.04** |
| **SD** | **912** | **35** | **39** | **0.007** | **0.015** | **23** | **28** | **0.01** | **0.02** |

*Low value may be caused by incomplete oral gavage.

**Table S5.** Plasma and brain concentrations (uncorrected and corrected), and concentration ratios of GNE-317 at 2 weeks and 6 weeks after the last radiation treatment (4 Gy x 10) in mice.

| **GNE-317** | | | | | | | | | |
| --- | --- | --- | --- | --- | --- | --- | --- | --- | --- |
|  | **Plasma (ng/mL)** | **Without correction** | | | | **Corrected for brain vasculature (1.4% of brain weight)** | | | |
|  |  | **Concentration (ng/g brain)** | | **Brain-to-plasma ratio** | | **Concentration (ng/g brain)** | | **Brain-to-plasma ratio** | |
|  |  | **Non-irradiated** | **Irradiated** | **Non-irradiated** | **Irradiated** | **Non-irradiated** | **Irradiated** | **Non-irradiated** | **Irradiated** |
| RT (4 Gy x 10) (2w) (n=5) | 3855 | 2148 | 2398 | 0.56 | 0.62 | 2094 | 2344 | 0.54 | 0.61 |
|  | 4984 | 2492 | 2599 | 0.50 | 0.52 | 2422 | 2529 | 0.49 | 0.51 |
|  | 4172 | 2334 | 2320 | 0.56 | 0.56 | 2275 | 2262 | 0.55 | 0.54 |
|  | 4158 | 2530 | 2395 | 0.61 | 0.58 | 2472 | 2337 | 0.59 | 0.56 |
|  | 4227 | 2129 | 2151 | 0.50 | 0.51 | 2070 | 2091 | 0.49 | 0.49 |
| **Average** | **4279** | **2327** | **2373** | **0.55** | **0.56** | **2267** | **2313** | **0.53** | **0.54** |
| **SD** | **420** | **187** | **161** | **0.05** | **0.05** | **184** | **158** | **0.05** | **0.05** |
| RT (4 Gy x 10) (6w) (n=5) | 1975 | 2137 | 1620 | 1.08 | 0.82 | 2109 | 1592 | 1.07 | 0.81 |
|  | 3413 | 2323 | 2309 | 0.68 | 0.68 | 2276 | 2261 | 0.67 | 0.66 |
|  | 3281 | 2649 | 2322 | 0.81 | 0.71 | 2603 | 2276 | 0.79 | 0.69 |
|  | 3351 | 2870 | 2243 | 0.86 | 0.67 | 2823 | 2196 | 0.84 | 0.66 |
|  | 3020 | 2501 | 2144 | 0.83 | 0.71 | 2458 | 2102 | 0.81 | 0.70 |
| **Average** | **3008** | **2496** | **2128** | **0.85** | **0.72** | **2454** | **2085** | **0.84** | **0.70** |
| **SD** | **597** | **284** | **292** | **0.15** | **0.06** | **278** | **284** | **0.15** | **0.06** |

**Table S6.** Plasma and brain concentrations (uncorrected and corrected), and concentration ratios of apitolisib at 2 weeks and 6 weeks after the last radiation treatment (4 Gy x 10) in mice.

| **Apitolisib** | | | | | | | | | |
| --- | --- | --- | --- | --- | --- | --- | --- | --- | --- |
|  | **Plasma (ng/mL)** | **Without correction** | | | | **Corrected for brain vasculature (1.4% of brain weight)** | | | |
|  |  | **Concentration (ng/g brain)** | | **Brain-to-plasma ratio** | | **Concentration (ng/g brain)** | | **Brain-to-plasma ratio** | |
|  |  | **Non-irradiated** | **Irradiated** | **Non-irradiated** | **Irradiated** | **Non-irradiated** | **Irradiated** | **Non-irradiated** | **Irradiated** |
| RT (4 Gy x 10) (2w) (n=5) | 8567 | 629 | 809 | 0.073 | 0.094 | 509 | 689 | 0.06 | 0.08 |
|  | 2209 | 221 | 230 | 0.100 | 0.104 | 191 | 199 | 0.09 | 0.09 |
|  | 1349 | 120 | 249 | 0.089 | 0.185 | 101 | 230 | 0.08 | 0.17 |
|  | 2025 | 166 | 136 | 0.082 | 0.067 | 138 | 107 | 0.07 | 0.05 |
|  | 4146 | 252 | 278 | 0.061 | 0.067 | 194 | 220 | 0.05 | 0.05 |
| **Average** | **3659** | **278** | **340** | **0.081** | **0.103** | **226** | **289** | **0.07** | **0.09** |
| **SD** | **2934** | **203** | **267** | **0.015** | **0.048** | **162** | **229** | **0.02** | **0.05** |
| RT (4 Gy x 10) (6w) (n=5) | 973 | 161 | 172 | 0.165 | 0.177 | 147 | 159 | 0.15 | 0.16 |
|  | 1312 | 138 | 153 | 0.105 | 0.117 | 120 | 134 | 0.09 | 0.10 |
|  | 928 | 171 | 88 | 0.184 | 0.095 | 158 | 75 | 0.17 | 0.08 |
|  | 923 | 559 | 187 | 0.606 | 0.203 | 547 | 174 | 0.59 | 0.19 |
|  | 2037 | 974 | 113 | 0.478 | 0.055 | 946 | 84 | 0.46 | 0.04 |
| **Average** | **1234** | **401** | **143** | **0.308** | **0.129** | **383** | **125** | **0.29** | **0.12** |
| **SD** | **477** | **365** | **41** | **0.221** | **0.060** | **360** | **44** | **0.22** | **0.06** |

**Table S7.** Plasma and brain concentrations (uncorrected and corrected), and concentration ratios of GNE-317 at 17, 65, 113, and 161 hours after 40 Gy x 1 hemi-brain irradiation in mice.

| **GNE-317** | | | | | | | | | |
| --- | --- | --- | --- | --- | --- | --- | --- | --- | --- |
|  | **Plasma (ng/mL)** | **Without correction** | | | | **Corrected for brain vasculature (1.4% of brain weight)** | | | |
|  |  | **Concentration (ng/g brain)** | | **Brain-to-plasma ratio** | | **Concentration (ng/g brain)** | | **Brain-to-plasma ratio** | |
|  |  | **Non-irradiated** | **Irradiated** | **Non-irradiated** | **Irradiated** | **Non-irradiated** | **Irradiated** | **Non-irradiated** | **Irradiated** |
| RT (40 Gy x 1) (17 hr) (n=3) | 2191 | 2452 | 2653 | 1.12 | 1.21 | 2421 | 2623 | 1.10 | 1.20 |
|  | 2559 | 2668 | 3116 | 1.04 | 1.22 | 2633 | 3080 | 1.03 | 1.20 |
|  | 2048 | 2081 | 2620 | 1.02 | 1.28 | 2053 | 2591 | 1.00 | 1.26 |
| **Average** | **2266** | **2400** | **2796** | **1.06** | **1.24** | **2369** | **2765** | **1.05** | **1.22** |
| **SD** | **263** | **297** | **277** | **0.05** | **0.04** | **294** | **273** | **0.05** | **0.04** |
| RT (40 Gy x 1) (65 hr) (n=3) | 3077 | 3145 | 3317 | 1.02 | 1.08 | 3102 | 3274 | 1.01 | 1.06 |
|  | 3233 | 3378 | 4147 | 1.04 | 1.28 | 3332 | 4102 | 1.03 | 1.27 |
|  | 3105 | 3524 | 3856 | 1.13 | 1.24 | 3481 | 3812 | 1.12 | 1.23 |
| **Average** | **3139** | **3349** | **3773** | **1.07** | **1.20** | **3305** | **3729** | **1.05** | **1.19** |
| **SD** | **83** | **191** | **421** | **0.06** | **0.11** | **191** | **420** | **0.06** | **0.11** |
| RT (40 Gy x 1) (113 hr) (n=3) | 1233 | 1303 | 1485 | 1.06 | 1.20 | 1285 | 1468 | 1.04 | 1.19 |
|  | 1937 | 2322 | 2179 | 1.20 | 1.12 | 2295 | 2152 | 1.18 | 1.11 |
|  | 429 | 2212 | 2615 | 5.15 | 6.09 | 2206 | 2609 | 5.14 | 6.08 |
| **Average** | **1200** | **1946** | **2093** | **2.47** | **2.81** | **1929** | **2076** | **2.46** | **2.79** |
| **SD** | **755** | **559** | **570** | **2.33** | **2.85** | **559** | **574** | **2.33** | **2.85** |
| RT (40 Gy x 1) (161 hr) (n=3) | 2222 | 2757 | 2669 | 1.24 | 1.20 | 2726 | 2638 | 1.23 | 1.19 |
|  | 2419 | 2391 | 3054 | 0.99 | 1.26 | 2357 | 3021 | 0.97 | 1.25 |
|  | 2145 | 2145 | 2398 | 1.00 | 1.12 | 2115 | 2368 | 0.99 | 1.10 |
| **Average** | **2262** | **2431** | **2707** | **1.08** | **1.19** | **2399** | **2676** | **1.06** | **1.18** |
| **SD** | **141** | **308** | **330** | **0.14** | **0.07** | **308** | **328** | **0.14** | **0.07** |

**Table S8.** Plasma and brain concentrations (uncorrected and corrected), and concentration ratios of apitolisib at 17, 65, 113, and 161 hours after 40 Gy x 1 hemi-brain irradiation in mice.

| **Apitolisib** | | | | | | | | | |
| --- | --- | --- | --- | --- | --- | --- | --- | --- | --- |
|  | **Plasma (ng/mL)** | **Without correction** | | | | **Corrected for brain vasculature (1.4% of brain weight)** | | | |
|  |  | **Concentration (ng/g brain)** | | **Brain-to-plasma ratio** | | **Concentration (ng/g brain)** | | **Brain-to-plasma ratio** | |
|  |  | **Non-irradiated** | **Irradiated** | **Non-irradiated** | **Irradiated** | **Non-irradiated** | **Irradiated** | **Non-irradiated** | **Irradiated** |
| RT (40 Gy x 1) (17 hr) (n=3) | 2244 | 162 | 161 | 0.072 | 0.072 | 131 | 130 | 0.06 | 0.06 |
|  | 2260 | 169 | 210 | 0.075 | 0.093 | 138 | 178 | 0.06 | 0.08 |
|  | 1815 | 142 | 151 | 0.078 | 0.083 | 116 | 126 | 0.06 | 0.07 |
| **Average** | **2106** | **158** | **174** | **0.075** | **0.083** | **128** | **145** | **0.06** | **0.07** |
| **SD** | **252** | **14** | **31** | **0.003** | **0.011** | **11** | **29** | **0.00** | **0.01** |
| RT (40 Gy x 1) (65 hr) (n=3) | 2558 | 129 | 160 | 0.050 | 0.063 | 93 | 124 | 0.04 | 0.05 |
|  | 2295 | 182 | 194 | 0.079 | 0.084 | 150 | 161 | 0.07 | 0.07 |
|  | 2272 | 169 | 192 | 0.074 | 0.084 | 137 | 160 | 0.06 | 0.07 |
| **Average** | **2375** | **160** | **182** | **0.068** | **0.077** | **127** | **149** | **0.05** | **0.06** |
| **SD** | **159** | **28** | **19** | **0.016** | **0.013** | **30** | **21** | **0.02** | **0.01** |
| RT (40 Gy x 1) (113 hr) (n=3) | 4008 | 242 | 282 | 0.060 | 0.070 | 186 | 226 | 0.05 | 0.06 |
|  | 1397 | 95 | 97 | 0.068 | 0.069 | 75 | 77 | 1.18 | 1.11 |
|  | 240 | 55 | 78 | 0.229 | 0.325 | 52 | 75 | 5.14 | 6.08 |
| **Average** | **1882** | **131** | **152** | **0.119** | **0.155** | **104** | **126** | **2.12** | **2.42** |
| **SD** | **1930** | **98** | **113** | **0.095** | **0.147** | **72** | **86** | **2.67** | **3.22** |
| RT (40 Gy x 1) (161 hr) (n=3) | 3793 | 329 | 311 | 0.087 | 0.082 | 276 | 258 | 0.07 | 0.07 |
|  | 2286 | 171 | 192 | 0.075 | 0.084 | 139 | 160 | 0.06 | 0.07 |
|  | 2842 | 182 | 213 | 0.064 | 0.075 | 143 | 173 | 0.05 | 0.06 |
| **Average** | **2974** | **227** | **239** | **0.075** | **0.080** | **186** | **197** | **0.06** | **0.07** |
| **SD** | **762** | **88** | **64** | **0.011** | **0.005** | **78** | **53** | **0.01** | **0.00** |

**Table S9.** Plasma and brain concentrations (uncorrected and corrected), and concentration ratios of nedisertib at 2 and 5 hours after the last sham or radiation treatment (6 Gy x 5) in mice.

| **Nedisertib** | | | | | | | | | |
| --- | --- | --- | --- | --- | --- | --- | --- | --- | --- |
|  | **Plasma (ng/mL)** | **Without correction** | | | | **Corrected for brain vasculature (1.4% of brain weight)** | | | |
|  |  | **Concentration (ng/g brain)** | | **Brain-to-plasma ratio** | | **Concentration (ng/g brain)** | | **Brain-to-plasma ratio** | |
|  |  | **Non-irradiated** | **Irradiated** | **Non-irradiated** | **Irradiated** | **Non-irradiated** | **Irradiated** | **Non-irradiated** | **Irradiated** |
| 2 hr (n=4) | 3508 | 172 | 272 | 0.049 | 0.078 | 83.1 | 183.1 | 0.02 | 0.05 |
|  | 1784 | 88 | 93 | 0.049 | 0.052 | 42.2 | 47.4 | 0.02 | 0.03 |
|  | 1744 | 81 | 126 | 0.046 | 0.072 | 36.4 | 81.2 | 0.02 | 0.05 |
|  | 3808 | 225 | 352 | 0.059 | 0.092 | 127.9 | 254.7 | 0.03 | 0.07 |
| **Average** | **2711** | **141** | **211** | **0.051** | **0.074** | **72.4** | **141.6** | **0.03** | **0.05** |
| **SD** | **1100** | **69** | **122** | **0.006** | **0.017** | **42.4** | **94.9** | **0.01** | **0.02** |
| 5 hr (n=4) | 407 | 29 | 40 | 0.072 | 0.098 | 18.9 | 29.7 | 0.05 | 0.07 |
|  | 982 | 50 | 52 | 0.051 | 0.053 | 24.6 | 27.0 | 0.03 | 0.03 |
|  | 2663 | 123 | 202 | 0.046 | 0.076 | 55.4 | 134.2 | 0.02 | 0.05 |
|  | 236 | 24 | 26 | 0.102 | 0.112 | 18.0 | 20.4 | 0.08 | 0.09 |
| **Average** | **1072** | **57** | **80** | **0.068** | **0.085** | **29.2** | **52.8** | **0.04** | **0.06** |
| **SD** | **1108** | **46** | **82** | **0.025** | **0.026** | **17.7** | **54.4** | **0.03** | **0.03** |

**Table S10.** Plasma and brain concentrations (uncorrected and corrected), and concentration ratios of brigimadlin at 24 and 72 hours after the last sham or radiation treatment (6 Gy x 5) in mice.

| **Brigimadlin** | | | | | | | | | |
| --- | --- | --- | --- | --- | --- | --- | --- | --- | --- |
|  | **Plasma (ng/mL)** | **Without correction** | | | | **Corrected for brain vasculature (1.4% of brain weight)** | | | |
|  |  | **Concentration (ng/g brain)** | | **Brain-to-plasma ratio** | | **Concentration (ng/g brain)** | | **Brain-to-plasma ratio** | |
|  |  | **Non-irradiated** | **Irradiated** | **Non-irradiated** | **Irradiated** | **Non-irradiated** | **Irradiated** | **Non-irradiated** | **Irradiated** |
| 24 hr (n=5) | 5978 | 68 | 97 | 0.011 | 0.016 | 0.0 | 13.5 | 0.000 | 0.002 |
|  | 3463 | 68 | 108 | 0.019 | 0.031 | 19.0 | 59.2 | 0.005 | 0.017 |
|  | 4627 | 108 | 87 | 0.023 | 0.019 | 43.5 | 22.2 | 0.009 | 0.005 |
|  | 4367 | 91 | 108 | 0.021 | 0.025 | 29.8 | 46.6 | 0.007 | 0.011 |
|  | 3095 | 104 | 68 | 0.033 | 0.022 | 60.2 | 24.8 | 0.019 | 0.008 |
| **Average** | **4306** | **88** | **94** | **0.022** | **0.023** | **30.5** | **33.3** | **0.008** | **0.009** |
| **SD** | **1127** | **19** | **17** | **0.008** | **0.006** | **23.0** | **18.9** | **0.007** | **0.006** |
| 72 hr (n=5) | 5920 | 51 | 60 | 0.009 | 0.010 | 0.0 | 0.0 | 0.000 | 0.000 |
|  | 3197 | 66 | 89 | 0.021 | 0.028 | 21.5 | 44.6 | 0.007 | 0.014 |
|  | 4615 | 82 | 125 | 0.018 | 0.027 | 17.0 | 60.2 | 0.004 | 0.013 |
|  | 4106 | 30 | 86 | 0.007 | 0.021 | 0.0 | 28.9 | 0.000 | 0.007 |
|  | 1846 | 31 | 70 | 0.017 | 0.038 | 5.4 | 44.1 | 0.003 | 0.024 |
| **Average** | **3937** | **52** | **86** | **0.014** | **0.025** | **8.8** | **35.6** | **0.003** | **0.012** |
| **SD** | **1528** | **22** | **25** | **0.006** | **0.010** | **10.0** | **22.7** | **0.003** | **0.009** |

**Table S11.** Plasma and brain concentrations (uncorrected and corrected), and concentration ratios of brigimadlin at 15 and 28 days after the last sham or radiation treatment (6 Gy x 5) in mice.

| **Brigimadlin** | | | | | | | | | | |
| --- | --- | --- | --- | --- | --- | --- | --- | --- | --- | --- |
|  | **Non-irradiated Plasma (ng/mL)** | **Irradiated Plasma (ng/mL)** | **Without correction** | | | | **Corrected for brain vasculature (1.4% of brain weight)** | | | |
|  |  |  | **Concentration (ng/g brain)** | | **Brain-to-plasma ratio** | | **Concentration (ng/g brain)** | | **Brain-to-plasma ratio** | |
|  |  |  | **Non-irradiated** | **Irradiated** | **Non-irradiated** | **Irradiated** | **Non-irradiated** | **Irradiated** | **Non-irradiated** | **Irradiated** |
| 15 days (n=5) | 4071 | 3832 | 123 | 125 | 0.030 | 0.032 | 65.7 | 70.9 | 0.016 | 0.018 |
|  | 6388 | 2690 | 103 | 94 | 0.016 | 0.035 | 13.2 | 56.5 | 0.002 | 0.021 |
|  | 4282 | 5225 | 95 | 134 | 0.022 | 0.026 | 35.2 | 60.7 | 0.008 | 0.012 |
|  | 4825 | 2690 | 141 | 89 | 0.029 | 0.033 | 73.5 | 51.1 | 0.015 | 0.019 |
|  | 4247 | 4114 | 119 | 136 | 0.028 | 0.033 | 59.0 | 78.0 | 0.014 | 0.019 |
| **Average** | **4763** | **3710** | **116** | **115** | **0.025** | **0.032** | **49.3** | **63.4** | **0.011** | **0.018** |
| **SD** | **951** | **1067** | **18** | **22** | **0.006** | **0.004** | **24.8** | **10.9** | **0.006** | **0.004** |
| 28 days (n=5) | 1291 | 3522 | 35 | 75 | 0.027 | 0.021 | 17.0 | 25.4 | 0.013 | 0.007 |
|  | 1721 | 3891 | 49 | 83 | 0.029 | 0.021 | 25.1 | 28.6 | 0.015 | 0.007 |
|  | 2534 | 1126 | 74 | 46 | 0.029 | 0.040 | 38.0 | 29.8 | 0.015 | 0.026 |
|  | 1567 | 1218 | 47 | 50 | 0.030 | 0.041 | 25.2 | 33.3 | 0.016 | 0.027 |
|  | 1080 | 2129 | 33 | 90 | 0.031 | 0.042 | 17.9 | 60.2 | 0.017 | 0.028 |
| **Average** | **1639** | **2377** | **48** | **69** | **0.029** | **0.033** | **24.6** | **35.5** | **0.015** | **0.019** |
| **SD** | **558** | **1282** | **16** | **20** | **0.001** | **0.011** | **8.4** | **14.1** | **0.001** | **0.011** |

**Table S12.** Plasma and brain concentrations (uncorrected and corrected), and concentration ratios of brigimadlin at 94, 133 and 183 days after the last sham or radiation treatment (6 Gy x 5) in mice.

| **Brigimadlin** | | | | | | | | | | |
| --- | --- | --- | --- | --- | --- | --- | --- | --- | --- | --- |
|  | **Non-irradiated Plasma (ng/mL)** | **Irradiated Plasma (ng/mL)** | **Without correction** | | | | **Corrected for brain vasculature (1.4% of brain weight)** | | | |
|  |  |  | **Concentration (ng/g brain)** | | **Brain-to-plasma ratio** | | **Concentration (ng/g brain)** | | **Brain-to-plasma ratio** | |
|  |  |  | **Non-irradiated** | **Irradiated** | **Non-irradiated** | **Irradiated** | **Non-irradiated** | **Irradiated** | **Non-irradiated** | **Irradiated** |
| 94 days (n=4 in Sham; n=5 in RT) | 2574 | 2731 | 109 | 126 | 0.042 | 0.046 | 73.2 | 87.8 | 0.028 | 0.032 |
|  | 2651 | 3682 | 92 | 145 | 0.035 | 0.039 | 54.4 | 93.4 | 0.021 | 0.025 |
|  | 1292 | 2966 | 53 | 101 | 0.041 | 0.034 | 34.4 | 59.0 | 0.027 | 0.020 |
|  | 1458 | 4905 | 56 | 109 | 0.039 | 0.022 | 36.0 | 39.9 | 0.025 | 0.008 |
|  | no sample | 5900 | no sample | 144 | NA | 0.024 | no sample | 61.4 | NA | 0.010 |
| **Average** | **1994** | **4037** | **77** | **125** | **0.039** | **0.033** | **49.5** | **68.3** | **0.025** | **0.019** |
| **SD** | **718** | **1342** | **28** | **20** | **0.003** | **0.010** | **18.2** | **22.1** | **0.003** | **0.010** |
| 133 days (n=5) | 2615 | 2574 | 66 | 77 | 0.025 | 0.030 | 29.7 | 40.5 | 0.011 | 0.016 |
|  | 1406 | 2382 | 34 | 77 | 0.024 | 0.032 | 14.5 | 43.2 | 0.010 | 0.018 |
|  | 508 | 4702 | 14 | 111 | 0.028 | 0.024 | 7.0 | 45.5 | 0.014 | 0.010 |
|  | 3085 | 2736 | 88 | 83 | 0.028 | 0.030 | 44.7 | 44.5 | 0.014 | 0.016 |
|  | 3309 | 4226 | 81 | 79 | 0.024 | 0.019 | 34.7 | 20.0 | 0.010 | 0.005 |
| **Average** | **2185** | **3324** | **57** | **85** | **0.026** | **0.027** | **26.1** | **38.7** | **0.012** | **0.013** |
| **SD** | **1191** | **1062** | **32** | **15** | **0.002** | **0.006** | **15.3** | **10.6** | **0.002** | **0.006** |
| 183 days (n=4 in Sham; n=5 in RT) | 4758 | 2953 | 146 | 88 | 0.031 | 0.030 | 79.8 | 46.9 | 0.017 | 0.016 |
|  | 1505 | 4791 | 70 | 128 | 0.047 | 0.027 | 49.1 | 61.3 | 0.033 | 0.013 |
|  | 4288 | 7723 | 112 | 138 | 0.026 | 0.018 | 52.2 | 29.6 | 0.012 | 0.004 |
|  | 1945 | 3820 | 37 | 121 | 0.019 | 0.032 | 9.4 | 67.7 | 0.005 | 0.018 |
|  | no sample | 4539 | no sample | 159 | NA | 0.035 | no sample | 95.2 | NA | 0.021 |
| **Average** | **3124** | **4765** | **91** | **127** | **0.031** | **0.028** | **47.6** | **60.1** | **0.017** | **0.014** |
| **SD** | **1637** | **1801** | **48** | **26** | **0.012** | **0.007** | **29.0** | **24.5** | **0.012** | **0.007** |

NA: not applicable.

**Table S13.** Plasma, normal brain, and intracranial tumor concentrations (uncorrected and corrected), and concentration ratios of brigimadlin in orthotopic GBM108 PDX models after sham or radiation treatment (6 Gy x 5).

| **Brigimadlin in GBM108 (n=4 or 5)** | | | | | | | | | | |
| --- | --- | --- | --- | --- | --- | --- | --- | --- | --- | --- |
|  | **Non-irradiated Plasma (ng/mL)** | **Irradiated Plasma (ng/mL)** | **Without correction** | | | | **Corrected for brain vasculature (1.4% of tissue weight)** | | | |
|  |  |  | **Concentration (ng/g tissue)** | | **Tissue-to-plasma ratio** | | **Concentration (ng/g tissue)** | | **Tissue-to-plasma ratio** | |
|  |  |  | **Non-irradiated** | **Irradiated** | **Non-irradiated** | **Irradiated** | **Non-irradiated** | **Irradiated** | **Non-irradiated** | **Irradiated** |
| Brain | 571 | 1065 | 24.0 | 41.4 | 0.042 | 0.039 | 16.0 | 26.5 | 0.03 | 0.02 |
|  | 202 | 488 | 8.7 | 18.0 | 0.043 | 0.037 | 5.9 | 11.2 | 0.03 | 0.02 |
|  | 149 | 584 | 9.0 | 28.2 | 0.060 | 0.048 | 6.9 | 20.0 | 0.05 | 0.03 |
|  | 189 | 686 | 7.2 | 35.1 | 0.038 | 0.051 | 4.6 | 25.5 | 0.02 | 0.04 |
|  | no sample | 896 | no sample | 36.6 | NA | 0.041 | no sample | 24.1 | NA | 0.03 |
| **Average** | **278** | **744** | **12.2** | **31.9** | **0.046** | **0.043** | **8.3** | **21.4** | **0.03** | **0.03** |
| **SD** | **197** | **235** | **7.9** | **9.1** | **0.010** | **0.006** | **5.2** | **6.3** | **0.01** | **0.01** |
| Tumor | 571 | 1065 | 168.6 | 194.4 | 0.30 | 0.18 | 160.6 | 179.5 | 0.28 | 0.17 |
|  | 202 | 488 | 246.0 | 248.4 | 1.22 | 0.51 | 243.2 | 241.6 | 1.20 | 0.50 |
|  | 149 | 584 | 47.4 | 157.8 | 0.32 | 0.27 | 45.3 | 149.6 | 0.30 | 0.26 |
|  | 189 | 686 | 174.6 | 123.6 | 0.93 | 0.18 | 172.0 | 114.0 | 0.91 | 0.17 |
|  | no sample | 896 | no sample | 261.6 | NA | 0.29 | no sample | 249.1 | NA | 0.28 |
| **Average** | **278** | **744** | **159.2** | **197.2** | **0.69** | **0.29** | **155.3** | **186.7** | **0.68** | **0.27** |
| **SD** | **197** | **235** | **82.4** | **58.6** | **0.46** | **0.13** | **81.9** | **58.3** | **0.46** | **0.13** |

NA: not applicable.

**Table S14.** Plasma, normal brain, and intracranial tumor concentrations (uncorrected and corrected), and concentration ratios of nedisertib in orthotopic GBM108 PDX models after sham or radiation treatment (6 Gy x 5).

| **Nedisertib in GBM108 (n=4 or 5)** | | | | | | | | | | |
| --- | --- | --- | --- | --- | --- | --- | --- | --- | --- | --- |
|  | **Non-irradiated Plasma (ng/mL)** | **Irradiated Plasma (ng/mL)** | **Without correction** | | | | **Corrected for brain vasculature (1.4% of tissue weight)** | | | |
|  |  |  | **Concentration (ng/g tissue)** | | **Tissue-to-plasma ratio** | | **Concentration (ng/g tissue)** | | **Tissue-to-plasma ratio** | |
|  |  |  | **Non-irradiated** | **Irradiated** | **Non-irradiated** | **Irradiated** | **Non-irradiated** | **Irradiated** | **Non-irradiated** | **Irradiated** |
| Brain | 3130 | 4283 | 209.2 | 178.8 | 0.067 | 0.042 | 165.4 | 118.8 | 0.05 | 0.03 |
|  | 2700 | 3338 | 170.0 | 194.4 | 0.063 | 0.058 | 132.2 | 147.7 | 0.05 | 0.04 |
|  | 2638 | 1612 | no sample | 99.6 | NA | 0.062 | no sample | 77.0 | NA | 0.05 |
|  | 4097 | 2210 | 239.6 | 106.4 | 0.058 | 0.048 | 182.2 | 75.5 | 0.04 | 0.03 |
|  | 3097 | 2203 | 227.2 | 110.8 | 0.073 | 0.050 | 183.8 | 80.0 | 0.06 | 0.04 |
| **Average** | **3132** | **2729** | **211.5** | **138.0** | **0.065** | **0.052** | **165.9** | **99.8** | **0.05** | **0.04** |
| **SD** | **584** | **1070** | **30.4** | **44.9** | **0.006** | **0.008** | **24.0** | **32.2** | **0.01** | **0.01** |
| Tumor | 3130 | 4283 | 440.8 | 328.0 | 0.141 | 0.077 | 397.0 | 268.0 | 0.13 | 0.06 |
|  | 2700 | 3338 | 308.0 | 424.0 | 0.114 | 0.127 | 270.2 | 377.3 | 0.10 | 0.11 |
|  | 2638 | 1612 | no sample | 170.4 | NA | 0.106 | no sample | 147.8 | NA | 0.09 |
|  | 4097 | 2210 | 329.6 | 239.2 | 0.080 | 0.108 | 272.2 | 208.3 | 0.07 | 0.09 |
|  | 3097 | 2203 | 340.8 | 259.2 | 0.110 | 0.118 | 297.4 | 228.4 | 0.10 | 0.10 |
| **Average** | **3132** | **2729** | **354.8** | **284.2** | **0.111** | **0.107** | **309.2** | **246.0** | **0.10** | **0.09** |
| **SD** | **584** | **1070** | **58.9** | **96.3** | **0.025** | **0.019** | **59.8** | **85.3** | **0.02** | **0.02** |

NA: not applicable.

**Table S15.** Plasma, normal brain, and intracranial tumor concentrations (uncorrected and corrected), and concentration ratios of brigimadlin in orthotopic GBM10 PDX models after sham or radiation treatment (6 Gy x 5).

| **Brigimadlin in GBM108 (n=6 or 9)** | | | | | | | | | | |
| --- | --- | --- | --- | --- | --- | --- | --- | --- | --- | --- |
|  | **Non-irradiated Plasma (ng/mL)** | **Irradiated Plasma (ng/mL)** | **Without correction** | | | | **Corrected for brain vasculature (1.4% of tissue weight)** | | | |
|  |  |  | **Concentration (ng/g tissue)** | | **Tissue-to-plasma ratio** | | **Concentration (ng/g tissue)** | | **Tissue-to-plasma ratio** | |
|  |  |  | **Non-irradiated** | **Irradiated** | **Non-irradiated** | **Irradiated** | **Non-irradiated** | **Irradiated** | **Non-irradiated** | **Irradiated** |
| Brain | 755 | 554 | 30.8 | 24.4 | 0.041 | 0.044 | 20.2 | 16.6 | 0.027 | 0.030 |
|  | 354 | 922 | 18.4 | 44.4 | 0.052 | 0.048 | 13.4 | 31.5 | 0.038 | 0.034 |
|  | 1111 | 1040 | 60.0 | 48.0 | 0.054 | 0.046 | 44.4 | 33.4 | 0.040 | 0.032 |
|  | 287 | 461 | 16.0 | 26.0 | 0.056 | 0.056 | 12.0 | 19.6 | 0.042 | 0.042 |
|  | 874 | 428 | 47.6 | 23.6 | 0.054 | 0.055 | 35.4 | 17.6 | 0.040 | 0.041 |
|  | 269 | 891 | 17.2 | 37.6 | 0.064 | 0.042 | 13.4 | 25.1 | 0.050 | 0.028 |
|  | no sample | 669 | no sample | 31.2 | NA | 0.047 | no sample | 21.8 | NA | 0.033 |
|  | no sample | 587 | no sample | 34.8 | NA | 0.059 | no sample | 26.6 | NA | 0.045 |
|  | no sample | 868 | no sample | 47.6 | NA | 0.055 | no sample | 35.4 | NA | 0.041 |
| **Average** | **608** | **713** | **31.7** | **35.3** | **0.054** | **0.050** | **23.2** | **25.3** | **0.04** | **0.04** |
| **SD** | **354** | **222** | **18.4** | **9.7** | **0.007** | **0.006** | **13.6** | **7.0** | **0.01** | **0.01** |
| Tumor | 755 | 554 | 98.4 | 68.0 | 0.130 | 0.123 | 87.8 | 60.2 | 0.116 | 0.109 |
|  | 354 | 922 | 73.6 | 229.3 | 0.208 | 0.249 | 68.6 | 216.4 | 0.194 | 0.235 |
|  | 1111 | 1040 | 251.2 | no sample | 0.226 | NA | 235.6 | no sample | 0.212 | NA |
|  | 287 | 461 | 70.4 | 108.6 | 0.245 | 0.236 | 66.4 | 102.1 | 0.231 | 0.222 |
|  | 874 | 428 | 264.8 | no sample | 0.303 | NA | 252.6 | no sample | 0.289 | NA |
|  | 269 | 891 | BLQ | 128.3 | NA | 0.144 | BLQ | 115.9 | NA | 0.130 |
|  | no sample | 669 | no sample | 136.0 | NA | 0.203 | no sample | 126.6 | NA | 0.189 |
|  | no sample | 587 | no sample | 240.0 | NA | 0.409 | no sample | 231.8 | NA | 0.395 |
|  | no sample | 868 | no sample | 201.6 | NA | 0.232 | no sample | 189.4 | NA | 0.218 |
| **Average** | **608** | **713** | **151.7** | **158.8** | **0.223** | **0.228** | **142.2** | **148.9** | **0.21** | **0.21** |
| **SD** | **354** | **222** | **97.8** | **65.3** | **0.063** | **0.093** | **93.6** | **64.2** | **0.06** | **0.09** |

NA: not applicable.
